# Comparative phyloinformatics of virus genes at micro and macro levels in a distributed computing environment

**DOI:** 10.1186/1471-2105-9-S1-S23

**Published:** 2008-02-13

**Authors:** Dadabhai T Singh, Rahul Trehan, Bertil Schmidt, Timo Bretschneider

**Affiliations:** 1 Genvea Biosciences, 53 Craig Road, #04-01, Singapore 089691; 2 Nanyang Technological University, Nanyang Avenue N4-02a-32, Singapore 639798; 3 NICTA VRL, University of Melbourne, Parkville 3010, Australia

## Abstract

**Background:**

Preparedness for a possible global pandemic caused by viruses such as the highly pathogenic influenza A subtype H5N1 has become a global priority. In particular, it is critical to monitor the appearance of any new emerging subtypes. Comparative phyloinformatics can be used to monitor, analyze, and possibly predict the evolution of viruses. However, in order to utilize the full functionality of available analysis packages for large-scale phyloinformatics studies, a team of computer scientists, biostatisticians and virologists is needed – a requirement which cannot be fulfilled in many cases. Furthermore, the time complexities of many algorithms involved leads to prohibitive runtimes on sequential computer platforms. This has so far hindered the use of comparative phyloinformatics as a commonly applied tool in this area.

**Results:**

In this paper the graphical-oriented workflow design system called *Quascade *and its efficient usage for comparative phyloinformatics are presented. In particular, we focus on how this task can be effectively performed in a distributed computing environment. As a proof of concept, the designed workflows are used for the phylogenetic analysis of neuraminidase of H5N1 isolates (micro level) and influenza viruses (macro level). The results of this paper are hence twofold. Firstly, this paper demonstrates the usefulness of a graphical user interface system to design and execute complex distributed workflows for large-scale phyloinformatics studies of virus genes. Secondly, the analysis of neuraminidase on different levels of complexity provides valuable insights of this virus's tendency for geographical based clustering in the phylogenetic tree and also shows the importance of glycan sites in its molecular evolution.

**Conclusion:**

The current study demonstrates the efficiency and utility of workflow systems providing a biologist friendly approach to complex biological dataset analysis using high performance computing. In particular, the utility of the platform Quascade for deploying distributed and parallelized versions of a variety of computationally intensive phylogenetic algorithms has been shown. Secondly, the analysis of the utilized H5N1 neuraminidase datasets at macro and micro levels has clearly indicated a pattern of spatial clustering of the H5N1 viral isolates based on geographical distribution rather than temporal or host range based clustering.

## Background

Recent occurrences of pandemics like the *Severe Acute Respiratory Syndrome *(SARS) or *Avian Influenza *clearly underscore the threat and seriousness of global diseases. The steadily growing globalization makes it difficult to contain pandemics to a certain region. Therefore, pandemic control is of highest importance to human health. Unfortunately, the segmented nature of the genome of influenza viruses is very conducive for genetic shifts and their rapid spread across various genera augments genetic drift. For example, the human influenza pandemics in 1957 and 1968 were suggested to have been caused by re-assorted influenza viruses [1]. Moreover, the H5N1 outbreak in Hong Kong in 1997 has convincingly demonstrated the ability of an avian virus to make the transition from birds to humans directly without going through a perceived "permissible host". In particular, the H5N1 virus is believed to have acquired the *hemagglutinin *(HA) gene from A/goose/Guangdong/1/96 H5N1 and A/teal/Hong Kong/W312/97 H6N1, while the internal genes were received from A/quail/Hong Kong/G1/97 H2N2 or A/teal/Hong Kong/W312/97 H6N1, respectively [2], [3]. Even though this particular strain was eliminated by culling millions of chicken, its ancestors remains circulating in aquatic birds.

This paper proposes a new approach to pandemic control by constantly monitoring molecular evolution at both macro level (within the group of viruses) and micro level (within the group of strains) using comparative phyloinformatics. This can facilitate prediction of how these viruses are evolving in terms of spatial, temporal, and host dimensions, and therefore, allows for faster responses to new outbreaks as well as their diagnosis. However, corresponding phylogenetic tree construction algorithms suffer from long runtimes due to their high degrees of computational complexity as well as the large datasets involved. Therefore, it is necessary to develop informatics based solutions that use suitable algorithms and take advantage of distributed computing technologies to make such studies feasible in a reasonable amount of time. Furthermore, these solutions need to be integrated in a framework for pandemic control that is biologist-friendly. As a result, effective vaccines and antivirals can be designed more easily and within a shorter response time, since specific local strains can be targeted directly.

In this paper it is demonstrated how such a system can be developed using a new distributed workflow design system called *Quascade*. As a proof of concept, results for an actual systematic phyloinformatics analysis of *neuraminidase *(NA) genes of H5N1 isolates and influenza viruses are presented. Various phylogenetic algorithms; i.e. UPGMA, Neighbor-Joining, Maximum Parsimony, Maximum Likelihood and Mr. Bayes [4-7], are compared with respect to their efficiency and accuracy in deriving biologically meaningful results. In particular, the presented study illustrates how these algorithms can be integrated in a unique user-friendly workflow in order to enhance the efficiency of a comparative phyloinformatic analysis.

The selection of the receptor destroyer NA for this study is motivated by its following properties. NA belongs to the glycosyl hydrolase family of proteins and catalyzes the cleavage of sialic acid residues from viral and cellular glycoproteins. Its most important function is to remove the terminal sialic acids from HA in order to enable the virus budding. Moreover, it helps the virus spreading through the system by additionally removing sialic acids from cell surfaces. In particular, since variations in the NA gene can lead to the evolution of more potent strains, studying the molecular evolution of this gene using the latest algorithms and computing power is essential.

Several isolated studies have been conducted since the H5N1 outbreak in late 2003 to monitor the molecular evolution at gene and genome level [8], [9]. All these studies involve a phylogenetic analysis at one level or the other. However, no systematic efforts have been undertaken so far to evaluate the suitability of the used phylogenetic algorithms in yielding biologically meaningful results for the analysis of HA and NA gene products of H5N1 viruses in particular and influenza viruses in general.

## Results

**Figure 1:**
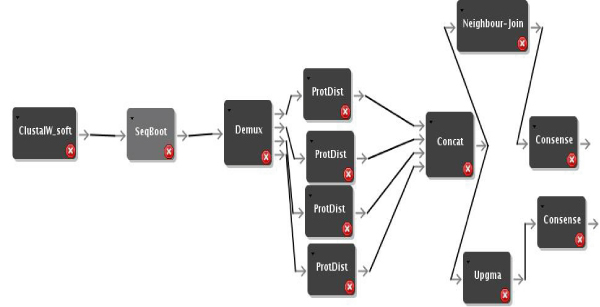
**Distributed Distance Workflow**. Distributed distance based workflow.

**Figure 2:**
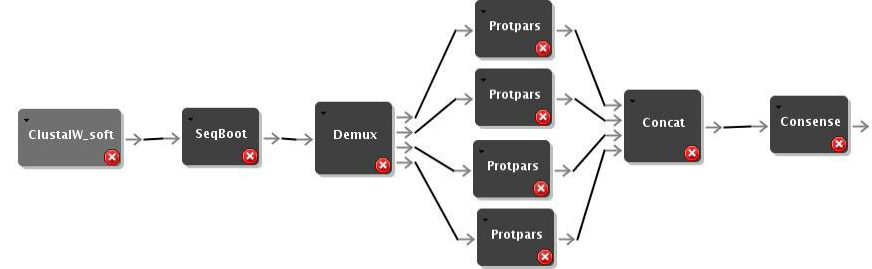
**Distributed Parsimony Workflow**. Distributed parsimony based workflow.

**Figure 3:**
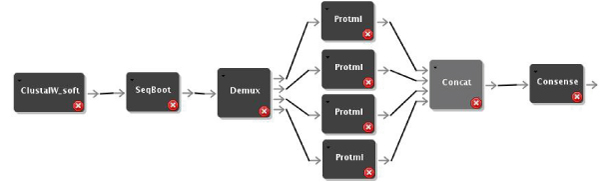
**Distributed ML Workflow**. Distributed ML based workflow.

We have used the new Quascade system to implement distributed workflows for comparative phyloinformatics using different algorithms. Figures 1, 2, and 3 show these workflows for distance-based algorithms, maximum parsimony algorithms and maximum likelihood algorithms, respectively. The workflows use ClustalW [10] to compute multiple sequence alignments and different phylogenetic algorithms from the PHYLIP package [4].

**Figure 4:**
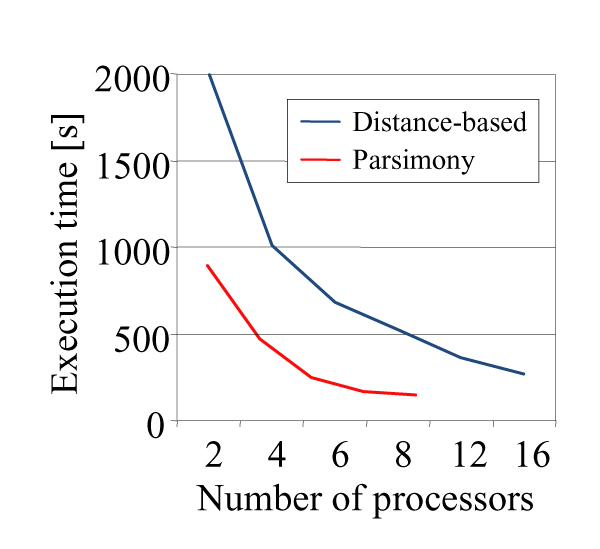
**Workflow Scalability**. Execution time vs. number of processors for 42 sequences and 96 data sets for distance and parsimony based workflows.

**Figure 5:**
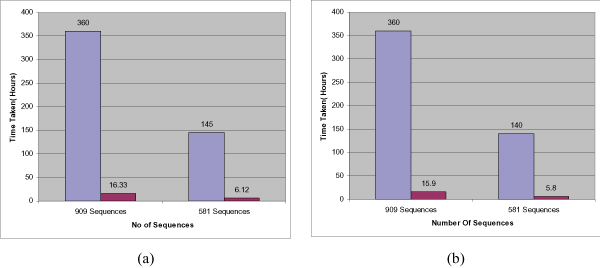
**Workflow Scalability for large data sets**. Execution time of the NA bird flu protein data sets consisting of 909 and 581 sequences respectively using 1 processor and 25 processors for (a) the distance-based workflow and (b) the parsimony workflow.

The most compute-intensive parts of each workflow (i.e. ProtDist in Figure 1, ProtPars in Figure 2, and ProtML in Figure 3) are executed in a distributed computing environment by simply multiplexing the data and using several instances of the respective programs. Figures 4 and 5 show execution times of the workflows for varying numbers of processors and sequences. In summary it can be seen that the system can achieve linear speedups.

We have used the designed workflows for the phyloinformatics analysis of NA in different populations of H5N1 in particular and in influenza viruses in general. The reason for choosing a protein-based phyloinformatics approach instead of a gene-based approach is to gain a better understanding of the molecular evolution of the gene product that makes this deadly virus spreading across different hosts. To perform this study, three protein data sets have been collected as follows.

1. A manual search of Swissprot [11] for NA and H5N1 has revealed only four entries: Q9WAA1  (A/Chicken/Hong Kong/220/1997 H5N1), Q710U6 (A/Chicken/Scotland/1959 H5N1), Q9Q0U7 (A/Goose/Guangdong/1/1996 H5N1), and Q9W7Y7 (A/Hong Kong/156/1997 H5N1).

2. A subsequent combined and refined search of Swissprot with TrEMBL (Translated European Molecular Biology Laboratory) has resulted in 18 more protein sequences. The resulting group of 22 NA sequences is used as the *core group*.

3. A *medium sized dataset *has been obtained comprising 581 entries pertaining to H5N1 NA that were mined from the Uniprot database [11].

4. A *macro dataset *of 909 entries of NA from all Influenza A viruses has been obtained from Uniprot.

These three datasets are shown in the additional files 1, 2, and 3.

**Figure 6:**
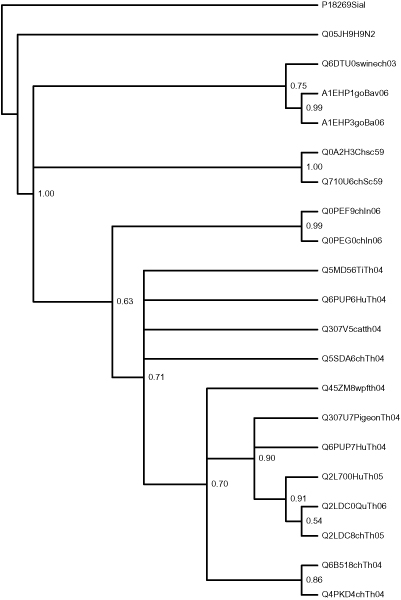
**Phylogram obtained from Mr. Bayes**. Phylograms obtained from Mr. Bayes for the dataset H5N1_NA_24.txt.

Phylograms obtained for the core group from the character based algorithms ProtPars [4] and ProtML [4] are shown in the additional files 4 and 5, while phylograms obtained from the two distance-based algorithms are shown in the additional files 6 and 7. The phylogram obtained by Mr. Bayes [7] is displayed in Figure 6. P18269Sial, a trypanosoan sialdase and Q05JH9H9N2, a NA of the distantly related H9N2 virus have been used as members of an outlier group for the phyloinformatics analysis.

**Figure 7:**
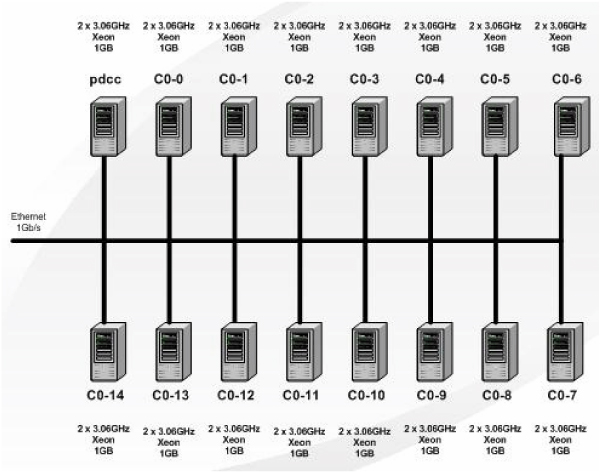
**PC cluster architecture**. The architecture of the utilized PC cluster.

Our phyloinformatics analysis with the core set has revealed a clear pattern: spatial clustering of the strains based on the particular geographical region rather than temporal clustering based on time scale or according to host range. The obtained trees for the medium-size data set are shown in the additional files 8, 9, and 10. The algorithms ProtPars, NJ, and UPGMA were used. All these algorithms have been distributed using the above Quascade workflows and deployed on a cluster of PCs. The utilized cluster consists of 16 nodes comprising 32 CPUs. Its detailed specification and architecture are shown in Table 1 and Figure 7, respectively. However, the algorithms ML and Mr. Bayes could not be run on the existing system with the medium dataset since it became apparent that these algorithms require further optimization in terms of distribution and/or more processing resources.

**Table 1:** Specification of PCs in the cluster.

**OS Platform**	**CPU Type**	**#CPU's**	**RAM**	**#PCs**
Rock 3.2.0 (2.4.21-15 ELsmp)	Intel Xeon 3.06 GHz	2	1 GB	16

Finally, in order to provide a global perspective of the molecular evolution of H5N1_NA, the dataset of 909 sequences of Influenza A viruses rather than H5N1 alone has been utilized. The objective of this macro level analysis is to signify the fact that this approach may lead to the nearest neighbors of some of the lethal clades which may be kept under observation for further evolution. Again, the large dataset was analyzed using the three algorithms ProtPars, NJ and UPGMA. The results obtained are shown in the additional files 11, 12, and 13. As per the core and medium sized datasets, the macro dataset also conforms to the clustering of H5N1_NA along the spatial lines rather than temporal or host range lines.

Keeping the Thai clade isolates in focus while analyzing the large dataset, the results obtained with ProtPars agree with the trend obtained so far. All twelve core sequences are mapped to the Thai clade. Besides these twelve, ten more sequences were found in this clade, whereby eight of them are fragments. The remaining two are actually complete sequences from Thailand (chicken isolates). However, they were annotated as *Neuramindase *instead of Neuraminidase [12], [13] which explains why these two entries are missing in the core set. However, querying of Uniprot with "Influenza virus" and "NA" as query terms has returned these two entries which are clearly captured and validated in our phyloinformatics analysis. Zooming out of the Thai clade has shown the Vietnam clade as its nearest neighbor, followed further by the Indian clade with Vietnam strains as their neighbors. On the next higher level Indonesian, Malaysian and Chinese clades are indicated as neighbors. It would be interesting to further study the Indian clade and verify how closely the Indian strains and Vietnamese strains are related with respect to other genes and also at genome level. Such an analysis may lead to the identification of the origin for Bird Flu in India. Further analysis of the large dataset indicates H9N2 as the nearest clade to H5N1 and non-structural protein as nearest to NA within the H5N1_NA group. The overall tree includes isolates from H3N2 to H15N9. A detailed analysis of each clade and sub-clade within this tree and with reference to the other two trees could lead to an understanding of H5N1's molecular evolution at global level.

## Discussion

The core set includes samples isolated over a time frame from 1959 to 2006 and represents globally distributed localities from Thailand to Bavaria, Germany. All the phylograms have essentially yielded geographic based clustering rather than time based clustering. The seven distinct clades that were clearly demarcated are: Thailand clade I (Q2L700; Q2LDC8; Q2LDC0; Q6PUP7; Q307U7; Q45ZM8), Thailand clade II (Q5SDA6; Q307V5; Q6PUP6; Q5MD56), Thailand clade III (Q6B518; Q4PKD4), Indian clade (Q0PEF9; Q0PEG0) Scotland clade (Q0A2H3; Q710U6) Bavarian clade (A1EHP1; A1EHP3), and China clade (Q6DTU0; Q9WAA1; Q9Q0U7; Q9W7Y7). It is interesting to note that the host range across these clades ranges from Chicken to Human consisting of 16 avian hosts and six mammalian hosts.

The clustering obtained using various algorithms has not shown any bias towards host based clustering. In fact, Thailand clade II consisted of a chicken, a cat, a tiger and a human. Multiple sequence alignments using ClustalW and rendered consecutively in BioEdit [24] have revealed the interesting and intriguing fact that the sequences of H5N1_NA from the same strain, which have infected all these hosts, are identical (see theadditional file 14). This observation indicates that even though NA is critical in spreading the virus to different hosts by means of its receptor destroying capacity, it may not be the sole factor for deciding the divergent host range. Accordingly a study was undertaken to monitor the phylogenetics of another critical protein, HA, and will be reported in a separate communication.

Multiple alignments of all 22 sequences have revealed another interesting fact. As can be seen from additional file 15, the earliest (in terms of the time scale) isolates that were considered belong to the Scotland clade which includes two isolates from chicken isolated in 1959. Both of them share an identical sequence homology. However, the even more important point to note here is the existence of a 20 amino acid stretch from position 48 to 68 in these clades which gets deleted in strains that evolved temporally. This stretch of twenty amino acids is absent in the isolates isolated from regions outside China and Hong Kong from 1996 onward. Another important observation to note is that the Goosander isolate from Guangdong province of China isolated in 1997 (Q9Q0U7/97) retained the same stretch of 20 amino acids as the Scotland clades but with more than 20 point mutations spread across the entire length. The deleted amino acid stretch included three N-glycosylation sites: NQSI, NNTW, and NQTY. The Chinese isolate (Q9Q0U7/97) retained all the glycosylation sites, whereas the two Hong Kong isolates have retained only the NQSI site but lost the other two. All the remaining 17 isolates have lost all the three glycosylation sites. Poon et al. [14] have shown the criticality of acquiring or losing glycan sites for effective viral spread in a study involving the phylogenetics of glycan interactions of the HIV envelop protein. A similar study is being undertaken by the authors of this work to evaluate the role of "lost" glycons in H5N1_NA.

A quick perusal of the results obtained with the three algorithms (ProtPars, NJ and UPGMA) has confirmed the pattern obtained from the core set. The strains of H5N1_NA cluster spatially rather than temporally or according to host. However, there are subtle differences amongst the outputs of the three algorithms with respect to their resolution. The clustering obtained using NJ seems to be better resolved than the other two in terms of branch length and sub-speciation. A detailed analysis of the Thai clade obtained by ProtPars also has revealed that all the sequences from the core set have been represented in this tree as well. The additional isolates are mostly fragments obtained through PCR amplification and uploaded to Uniprot. An interesting aspect of this clade is the finding that the entry Q5EP24 (Chicken isolate from Vietnam) is placed almost as an outlier in this group. It would be interesting to analyze the genealogies of Vietnam and Thailand clades and to verify whether there are any "bridging" isolates such as Q5EP24 that may play an important role in the spread of this deadly disease across the globe. We have analyzed our medium dataset to confirm the pattern we obtained with the core set. A detailed analysis of each geographic clade with respect to the "bridging" isolates such as Q5EP24 may reveal a global pattern of H5N1 spread.

## Conclusion

Global preparedness for H5N1 pandemic has been declared as the top priority of global health agencies such as CDC. In view of the ever escalating threat of the virus mutating to a human lethal form, it has become essential to constantly monitor the molecular evolution of this virus utilizing the latest and best phylogenetic algorithms.

The current study was undertaken with two objectives: One is to demonstrate the utility of a biologist's friendly high performance computing workflow system in analyzing large and complex biological datasets by deploying compute intensive algorithms in their parallelized version. The second objective is to analyze H5N1_NA data, as a proof of concept, in such a workflow to understand the molecular evolution of this rapidly evolving virus.

We have demonstrated the utility of workflow systems by designing a pipeline that starts with data input by the biologist. Once the relevant data is uploaded into the workflow the remaining steps are all automated. In particular these steps comprise starting with multiple alignments of sequences to the distribution of the output to various algorithms, distributing the relevant outputs to different servers in a distributed environment which is grid compatible, and finally visualizing the output. Such a workflow system saves a significant amount of time and eliminates possible human errors in analyzing critical data.

In any phylogenetic analysis involving pathogenic viruses there are three clear possibilities of clustering behavior: spatial, temporal and host based. Our analysis of H5N1 NA data utilizing different phylogenetic algorithms have indicated a spatial clustering of this virus based on geographical distribution rather than temporal or host. Of course, single gene product analysis is insufficient to arrive at any biologically relevant conclusion and, hence, we propose to do multiple gene and genome level analysis as our future work. However, even for a single gene analysis the computing power required is formidable and we have used this approach as a proof of the concept that high performance computing is a must for any meaningful phyloinformatics study. Even with the limited amount of data, we have been able to find a clear pattern (geographical clustering) and the importance of glycon sites. However, further detailed studies in conjunction with other proteins such as HA, Polymerase etc., and also at gene and genome level, are required to draw firm conclusions. It is also true that interpretation becomes more difficult if the size of the trees becomes very large. In our experience, zooming onto a cluster of interest (e.g. Thailand and China clades) with relative ease and quickness, using the Quascade middleware is an attractive feature of our study. Furthermore, the comparison of different algorithms simultaneously with the same input file is another attractive attribute.

## Methods

### Overview of Quascade-MP2

The exponential growth in the size of biological databases has established the need for high performance computing (HPC) in bioinformatics. Typically, an HPC setup operates in a cluster computing environment consisting of multiple computers that communicate over fast switches. For popular database scanning applications such as BLAST and HMMER the benefits of clusters are immediate and linear speedups can be easily achieved. However, the evolving challenges in life sciences cannot be all addressed by off-the-shelf bioinformatics applications. Life scientists need to analyze their data using novel approaches published in recent journals or based on their own hypotheses and assumptions. Quascade-MP2 has been developed to address this need. It is a visual prototyping tool created especially for data-driven, high performance scientific applications.

The complexity involved in using traditional technologies and tools often proves to be over-whelming and counter-productive. Most of these tools require an understanding of programming or scripting languages, such as Perl, Python, Java, and UNIX-scripts. Recent examples of such systems in bioinformatics include Biopipe [15], BioWBI [16], Taverna [17], Wildfire[18], KDE Biosciences [19], gRNA [20], Biowep [21], and BioWMS [22]. Each deployment is therefore subject to its own code development and testing. Although programming languages offer sophisticated control over the intended procedure, the learning curve and overheads in terms of human resources are difficult to justify. Quascade has been developed with this problem in mind.

Quascade has been designed for research applications characterized by strict high performance requirements. It provides a graphical, drag-and-drop interface to allow users to design and execute ad-hoc workflows. Workflows are constructed by an end-user by inter-connecting functional blocks, called *components*. A component is a piece of independent and self-contained code performing a given functionality on its input and generates an output. Data paths among components can be specified by drawing lines between/among the output(s) and input(s) of different components, respectively. Thereby, various combinations of these components (such as one output to multiple inputs, multiple outputs to multiple inputs) can be used to create any workflow depicting the flow of both data and logic.

**Figure 8:**
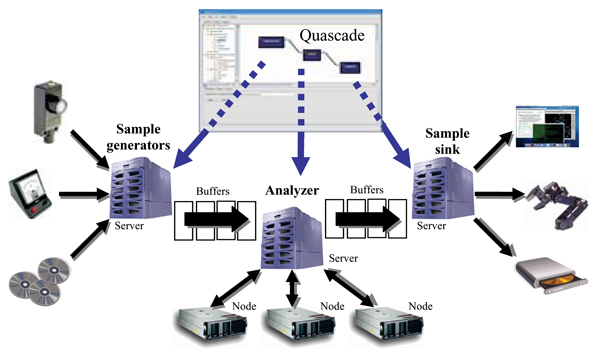
**Quascade-MP2 workflow**. Typical configuration for executing a Quascade-MP2 workflow

Individual components in a workflow may be designated to run on different computing servers in a cluster by simply specifying a corresponding condition in Quascade. This provides a straightforward approach of constructing workflows that execute in a high-performance environment. In turn, a component running on a given server may use several computing nodes to execute its program, thereby providing a two-tiered mechanism of distributed processing. As an example, Figure 8 shows a physical workflow and its graphical counterpart. The workflow consists of a *sample generator *component, which generates/collects data, an *analyzer *component which performs the actual processing, and a *sample sink *component which 'consumes' the processed data in terms of not forwarding the data further to another component. While the *generator *and *sink *components can be connected to external entities, i.e. physical devices, the *analyzer *component may be a simple single-node program or a complex, parallel multi-node application.

### Software/Hardware setup

A typical installation of Quascade with MP^2 ^middleware is installed on a centrally accessible network file system mount location, while a client uses Quascade to create and execute a workflow. Since each component in the workflow can be assigned to a particular MP^2 ^server, a straightforward implicit distribution can be achieved, where all communication among components is managed by Quascade and MP^2^. Complimentary, the workload distribution of an MP^2 ^server among its allocated MP^2 ^compute nodes is performed explicitly, i.e. it is the software developer's responsibility to parallelize a component in order to enable efficient simultaneous execution on multiple nodes.

### Communication issues

The user creates a workflow by selecting workflow components from a predefined list of deployed components on the cluster. Each component is configured to run on a particular cluster server or automatically assigned to a server if no explicit configuration is desired. At run-time Quascade performs a remote invocation to the selected servers and creates local instances of the used components. Hence, client-server communication takes place between a Quascade client and one or more instances of MP^2 ^servers forming the cluster. Complementary, server-server communication takes place between two or more servers or between a server and its compute nodes.

Two different types of communication can be differentiated: *explicit *and *implicit*. They distinguish between the workload distribution by a parallel component to multiple compute nodes and the communication between two of more servers exchanging data, respectively. Server-server communication uses raw sockets to transfer data from one machine to the other. This solution provides a performance benefit over more sophisticated alternatives such as RMI or CORBA, but represents the lowest level of abstraction of the underlying network. As an answer to this trade-off, MP^2 ^overcomes the limitations of development complexity and inflexibility by providing an abstraction at the component level supported by a fixed number of network level operations. Thus, a developer has a high degree of flexibility regarding the operation of a component as long as the implementation adheres to the I/O scheme provided by the communication layer. Implicit communication refers to the communication that takes place between the output of one component and the input of another component, i.e. the selected data paths among components. The underlying transfer mechanism is provided by the middleware and comprises input buffers in order to enable asynchronous communication. Input buffers are continuously polled by the corresponding component and processing continued once data is available. An adjustable buffer length according to load expectation helps to prevent overflows.

For example our workflow component for ClustalW shows all the options that the original ClustalW program presents to users. The options are presented in a parameter output panel and translated into command line string for calling the underlying ClustalW program upon execution of the workflow. After the completion of ClustalW, an output file is produced on the local hard disk at a specified location containing the aligned data set. This file name and its location is the input for the next component (e.g. Seqboot). The remaining components, e.g. Seqboot with its underlying PHYLIP operate in the same way.

**Figure 9:**
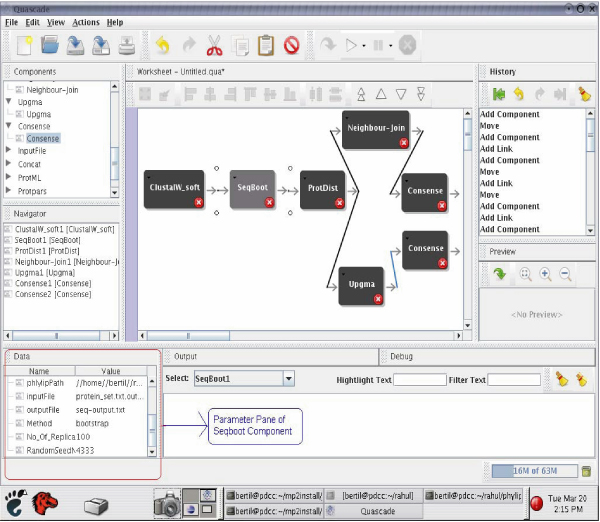
**Distance based Quascade workflow**. Screenshot of sequential distance based phylogenetic analysis workflow in Quascade.

For instance, Figure 9 shows a screenshot of the implemented sequential workflow for a distance based phylogenetic analysis.

**Figure 10:**
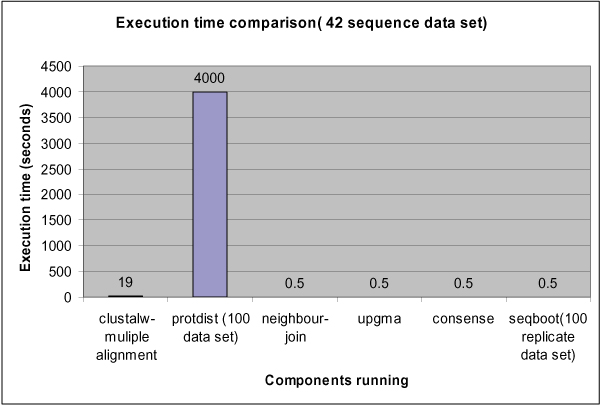
**Workflow Profiling**. Execution time of the distance based phylogenetic analysis workflow.

The runtime of this workflow has been profiled on a single server for an input of 42 sequences and *N *= 100 replica. As can be seen form Figure 10, the *Protdist *component clearly dominates the overall execution time and requires parallelization.

The Seqboot component is modified to break up its output file's data into several data sets, which are written to a buffered output port. Distribution of files is then implemented by using a demultiplexer (*Demux*) component and a result combining module (*Concat*). More technical details about the file distribution and concatenation as well as other workflow parameters can be found elsewhere [23]. The model, design and implementation used to distribute the distance-based workflow can also be applied to parallelize the parsimony and the ML based workflows. The usage of the model and design developed for the Protdist component has therefore been used for the Protpars and the ProtML components. The corresponding workflows are shown in Figure 1, 2, and 3.

## Competing interests

The authors declare that they have no competing interests.

## Authors' contributions

DTS, BS conceived the study. RT, DTS, BS performed computational studies and designed workflows. RT implemented the workflow software. DTS collected the data. DTS, BS, and TB contributed analyzing experimental studies. DTS, BS, RT and TB wrote the manuscript.

## Supplementary Material

Additional file 1 All sequence in the core data set. Click here for file

Additional file 2 All sequence in the medium data set. Click here for file

Additional file 3 All sequence in the macro data set. Click here for file

Additional file 4 Phylograms obtained from the Parsimony workflow for the dataset H5N1_NA_24.txt. Click here for file

Additional file 5 Phylograms obtained from the ML workflow for the dataset H5N1_NA_24.txt. Click here for file

Additional file 6 Phylograms obtained from the distance based workflow using UPGMA for the dataset H5N1_NA_24.txt. Click here for file

Additional file 7 Phylograms obtained from the distance workflow using NJ for the dataset H5N1_NA_24.txt. Click here for file

Additional file 8 Phylograms obtained from the distance workflow using UPGMA for the dataset H5N1_NA_medium.txt. Click here for file

Additional file 9 Phylograms obtained from the distance workflow using NJ for the dataset H5N1_NA_medium.txt. Click here for file

Additional file 10 Phylograms obtained from the parsimony workflow for the dataset H5N1_NA_medium.txt. Click here for file

Additional file 11 Phylograms obtained from the distance workflow using UPGMA for the dataset H5N1_NA_macro.txt. Click here for file

Additional file 12 Phylograms obtained from the distance workflow using NJ for the dataset H5N1_NA_macro.txt. Click here for file

Additional file 13 Phylograms obtained from the parsimony workflow for the dataset H5N1_NA_macro.txt. Click here for file

Additional file 14 Multiple Sequence Alignment of the Thailand clade II (Q5SDA6; Q307V5; Q6PUP6; Q5MD56) computed by ClustalW. Click here for file

Additional file 15 Multiple Sequence Alignment of all sequences in H5N1_NA_24.txt except the two outliers. Click here for file

## References

[B1] Zambon MC (2001). The pathogenesis of influenza in humans. Rev Med Virol.

[B2] Subbarao K, Shaw MW (2000). Molecular aspects of avian influenza (H5N1) viruses isolated from humans. Rev Med Virol.

[B3] Hatta M, Kawaoka Y (2002). The continued pandemic threat posed by avian influenza viruses in Hong Kong. Trends Microbiol.

[B4] PHYLIP Home Page. http://evolution.genetics.washington.edu/phylip.html.

[B5] Saitou N, Nei M (1987). The neighbor-joining method: A new method for reconstructing phylogenetic trees. Mol Biol Evol.

[B6] Holder M, Lewis PO (2003). Phylogeny estimation: Traditional and Bayesian approaches. Nature Reviews Genetics.

[B7] Ronquist F, Huelsenbeck JP (2003). MrBayes 3: Bayesian phylogenetic inference under mixed models. Bioinformatics.

[B8] Puthavathana P, Auewarakul P, Charoenying PC, Sangsiriwut K, Pooruk P, Boonnak K, Khanyok R, Thawachsupa P, Kijphati R, Sawanpanyalert P (2005). Molecular characterization of the complete genome of human influenza H5N1 virus isolates from Thailand. Journal of General Virology.

[B9] Tran TH, Nguyen TL, Nguyen TD, Luong TS, Pham PM, Nguyen VC, Pham TS, Vo CD, Le TQM, Ngo TT, Dao BK, Le PP, Nguyen TT, Hoang TL, Cao VT, Le TG, Nguyen DT, Le HN, Nguyen TKT, Le HS, Le VT, Dolecek C, Tran TT, de Jong M, Schultsz C, Cheng P, Lim W, Horby P, Farrar J, World Health Organization International Avian Influenza Investigative Team (2004). Avian influenza A (H5N1) in 10 patients in Vietnam. N Engl J Med.

[B10] Thompson JD, Higgins DG, Gibson TJ (1994). CLUSTAL W: Improving the sensitivity of progressive multiple sequence alignment through sequence weighting, position-specific gap penalties and weight matrix choice. Nucleic Acid Res.

[B11] ExPASy – UniProt Knowledgebase. http://expasy.org/sprot.

[B12] UniProtKB/TrEMBL entry Q6DPL8 [Q6DPL8_9INFA] Neuraminidase. Q6DPL8.

[B13] UniProtKB/TrEMBL entry Q6DPM0 [Q6DPM0_9INFA] Neuraminidase. Q6DPM0.

[B14] Poon AFY, Lewis F, Kosakovsky SL, Pond S, Frost DW Evolutionary interactions between N-linked glycosylation sites in the HIV envelope. PLoS Computational Biology.

[B15] Hoon S, Ratnapu KK, Chia J-M, Kumarasamy B, Xiao J, Clamp M, Stabenau A, Potter A, Clarke L, Stupka E (2003). Biopipe: A flexible framework for protocol-based bioinformatics analysis. Genome Research.

[B16] Leo P, Marinelli C, Pappadà G, Scioscia G, Zanchetta L (2004). BioWBI: An integrated tool for building and executing Bioinformatic analysis workflows. Proceedings of the Bioinformatics Italian Society Meeting.

[B17] Oinn T, Addis M, Ferris J, Marvin D, Senger M, Greenwood M, Carver T, Glover K, Pocock MR, Wipat A, Li P (2004). Taverna: A tool for the composition and enactment of bioinformatics workflows. Bioinformatics.

[B18] Tang F, Chua C, Ho L, Lim Y, Issac P, Krishnan A (2005). Wildfire: Distributed, Grid-enabled workflow construction and execution. BMC Bioinformatics.

[B19] Lua Q, Haob P, Curcinc V, Heb W, Lib Y-Y, Luoa Q-M, Guoc Y-K, Lib Y-X (2006). KDE Biosciences: Platform for bioinformatics analysis workflows. Journal of Biomedical Informatics.

[B20] Schmidt B, Lin F, Laud A, Santoso Y (2004). Development of distributed bioinformatics applications with GMP. Concurrency and Computation: Practice and Experience.

[B21] Romano P, Bartocci E, Bertolini G, De Paoli F, Marra D, Mauri G, Merelli E, Milanesi L (2007). Biowep: A workflow enactment portal for bioinformatics applications. BMC Bioinformatics.

[B22] Bartocci E, Corradini F, Merelli E, Scortichini L (2007). BioWMS: A web-based workflow management system for bioinformatics. BMC Bioinformatics.

[B23] Singh DT, Trehan R, Ray P, Schmidt B (2007). Phylogenetic analysis of neuraminidase genes of H5N1 isolates using HPC technologies. Proceedings of the IEEE International Conference on e-Health Networking, Application and Services.

[B24] BioEdit Sequence Alignment Editor for Windows 95/98/NT/XP. http://www.mbio.ncsu.edu/BioEdit/bioedit.html.

