# Supplementary figures and images for: Comparative phyloinformatics of virus genes at micro and macro levels in a distributed computing environment
Source: BMC Bioinformatics. 2008 Feb 13;9(Suppl 1):S23. doi: 10.1186/1471-2105-9-S1-S23 (PMC2259424; doi:10.1186/1471-2105-9-S1-S23)

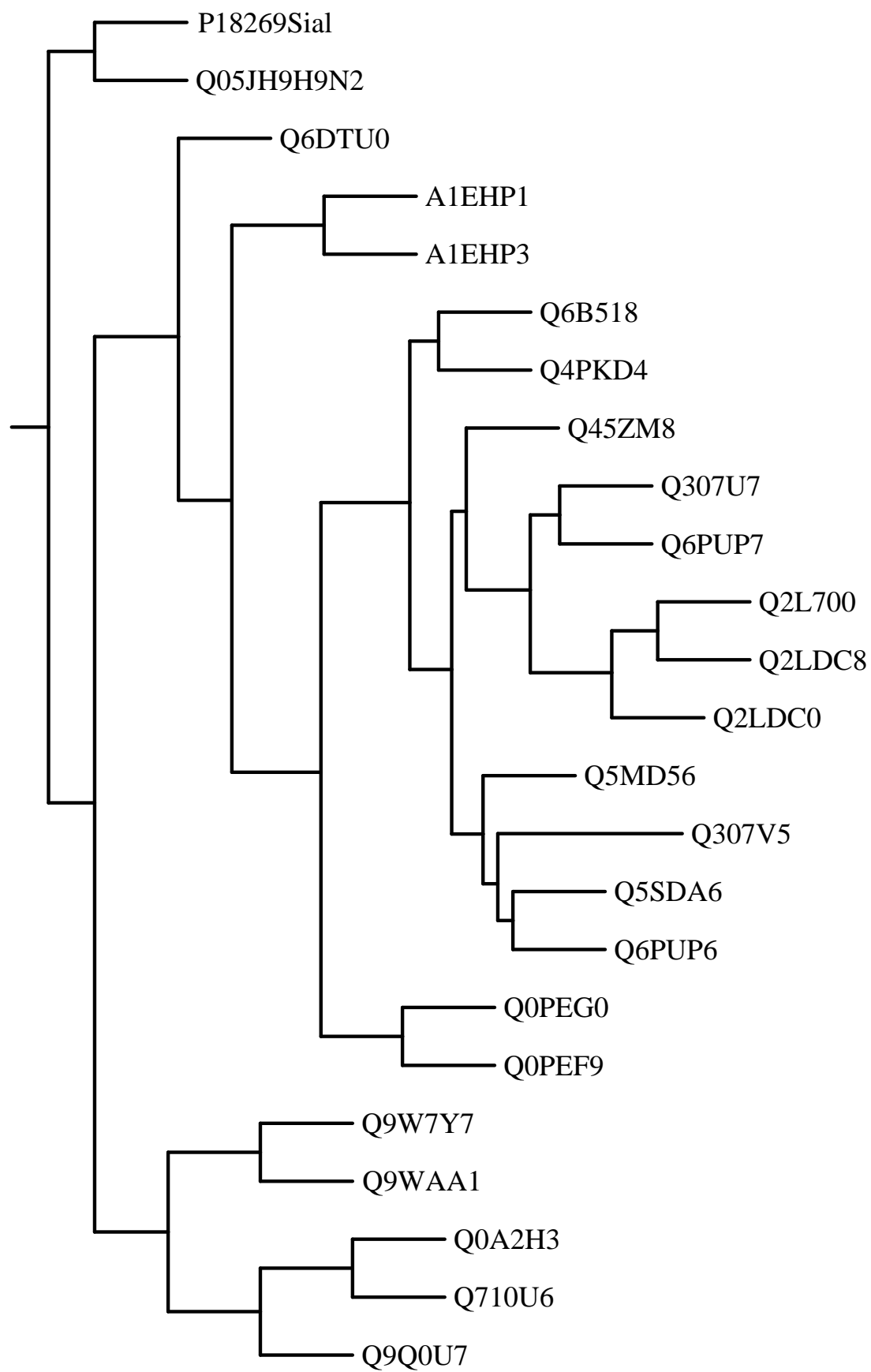

Supplement: Additional file 4 — Phylograms obtained from the Parsimony workflow for the dataset H5N1_NA_24.txt. [file 1471-2105-9-S1-S23-S4.pdf]

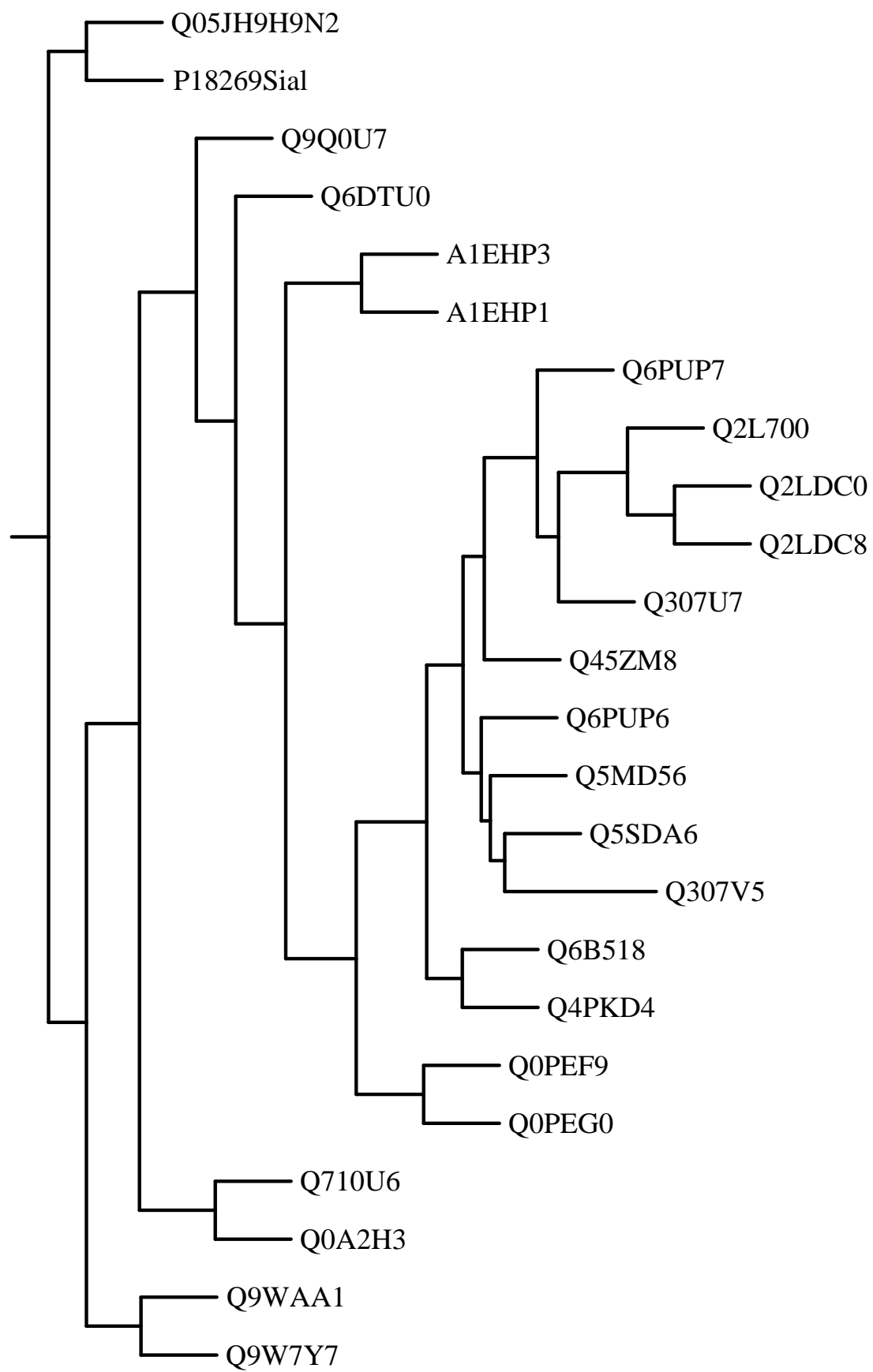

Supplement: Additional file 5 — Phylograms obtained from the ML workflow for the dataset H5N1_NA_24.txt. [file 1471-2105-9-S1-S23-S5.pdf]

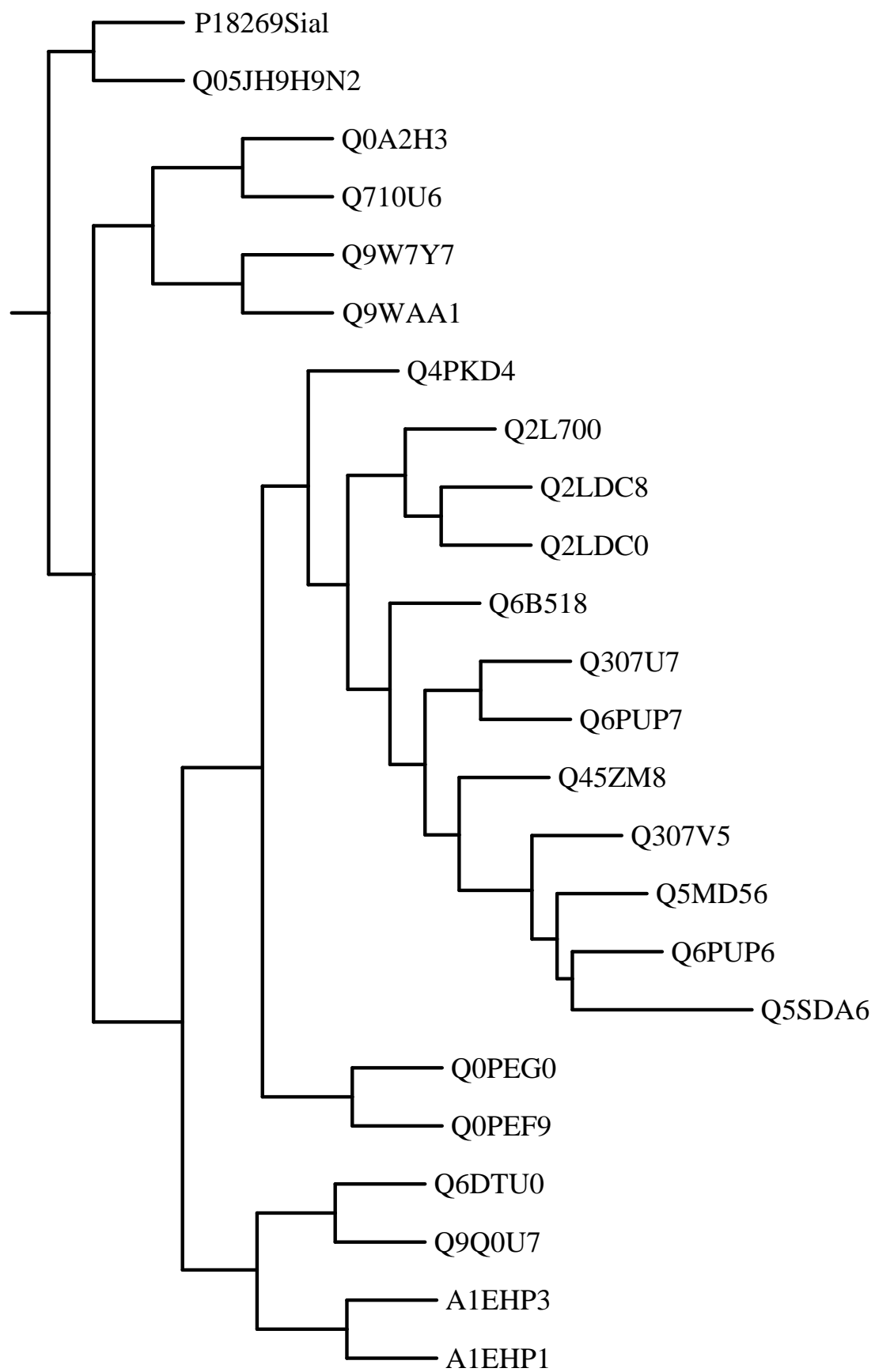

Supplement: Additional file 6 — Phylograms obtained from the distance based workflow using UPGMA for the dataset H5N1_NA_24.txt. [file 1471-2105-9-S1-S23-S6.pdf]

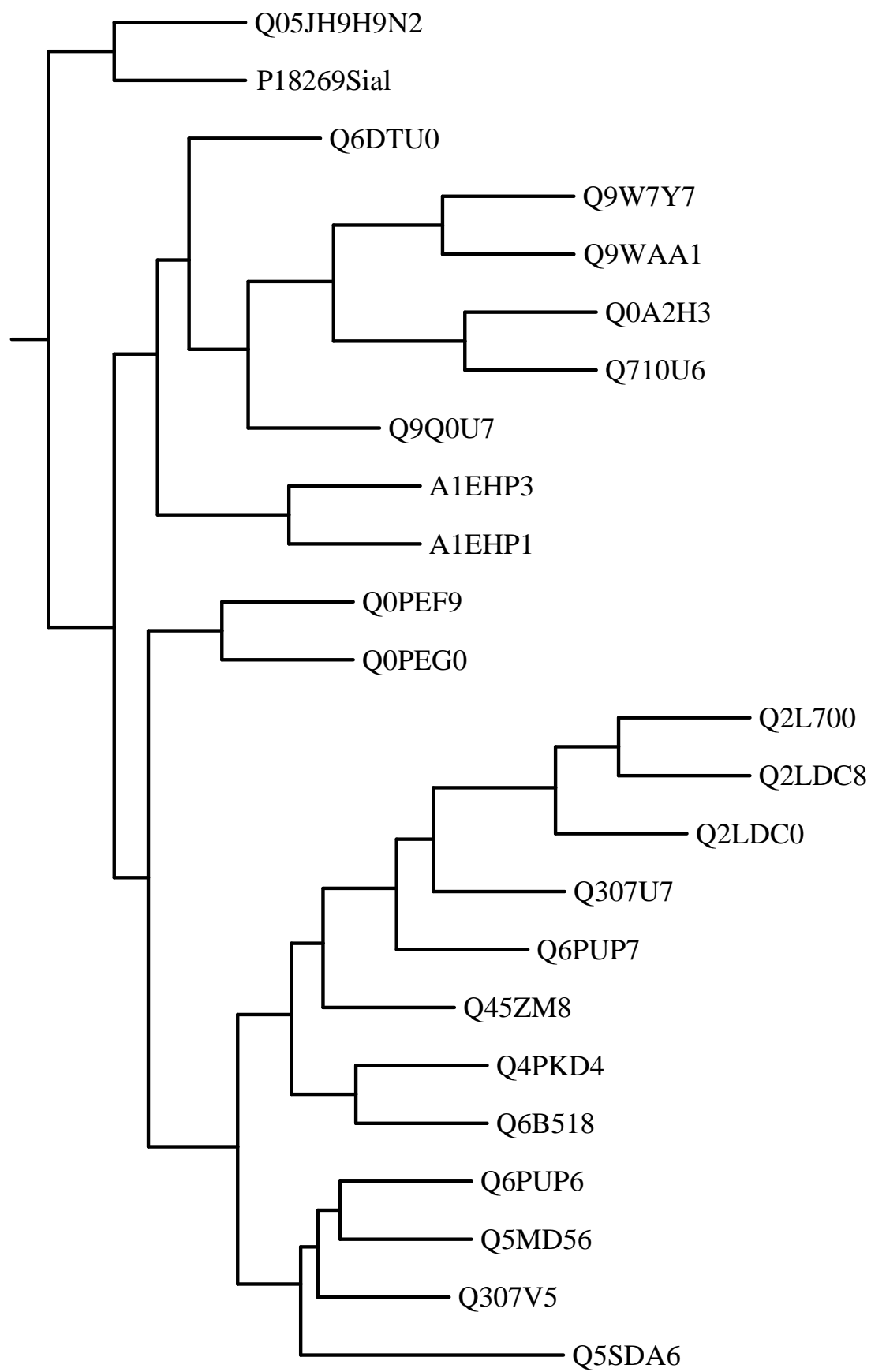

Supplement: Additional file 7 — Phylograms obtained from the distance workflow using NJ for the dataset H5N1_NA_24.txt. [file 1471-2105-9-S1-S23-S7.pdf]

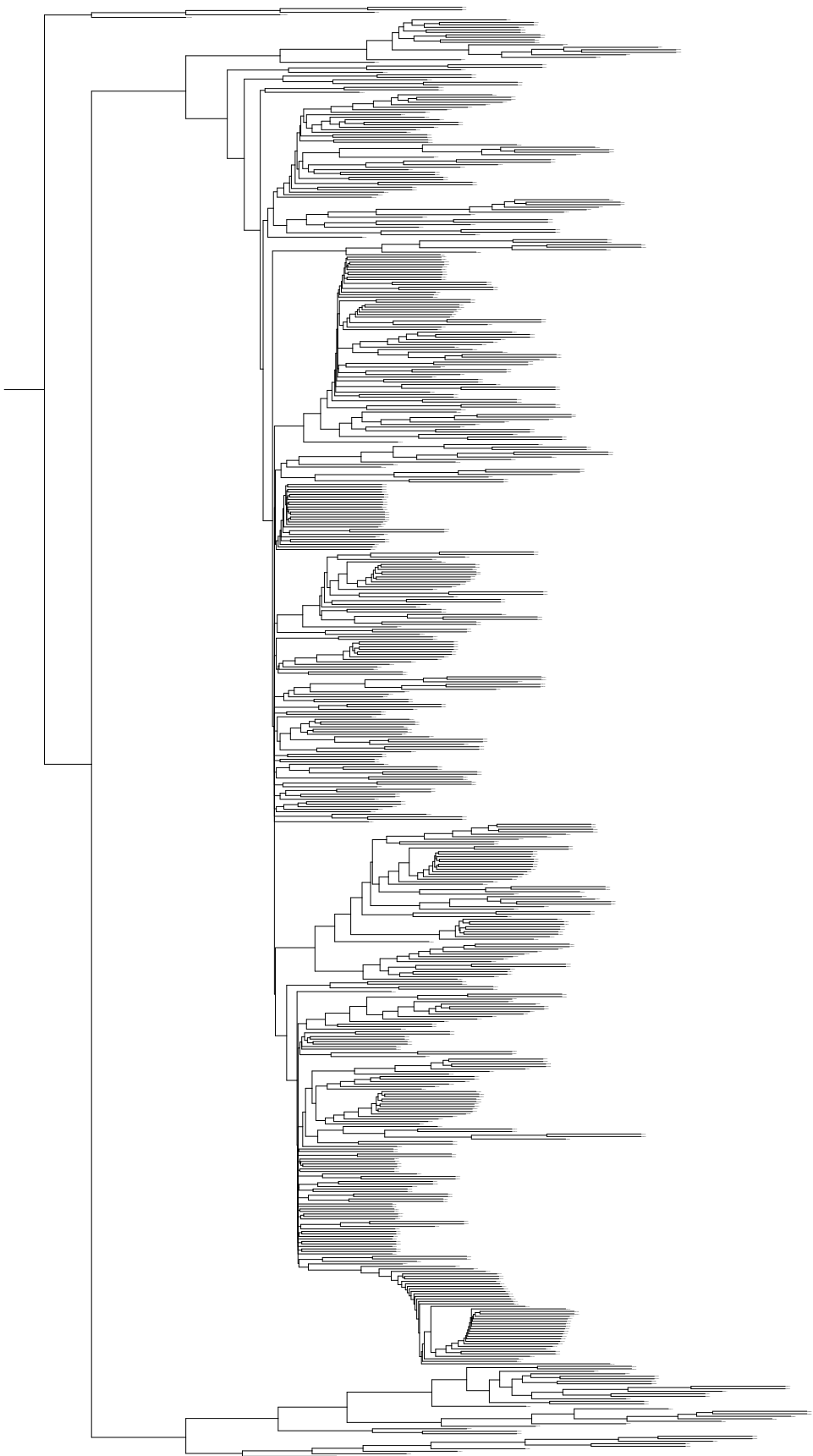

Supplement: Additional file 8 — Phylograms obtained from the distance workflow using UPGMA for the dataset H5N1_NA_medium.txt. [file 1471-2105-9-S1-S23-S8.pdf]

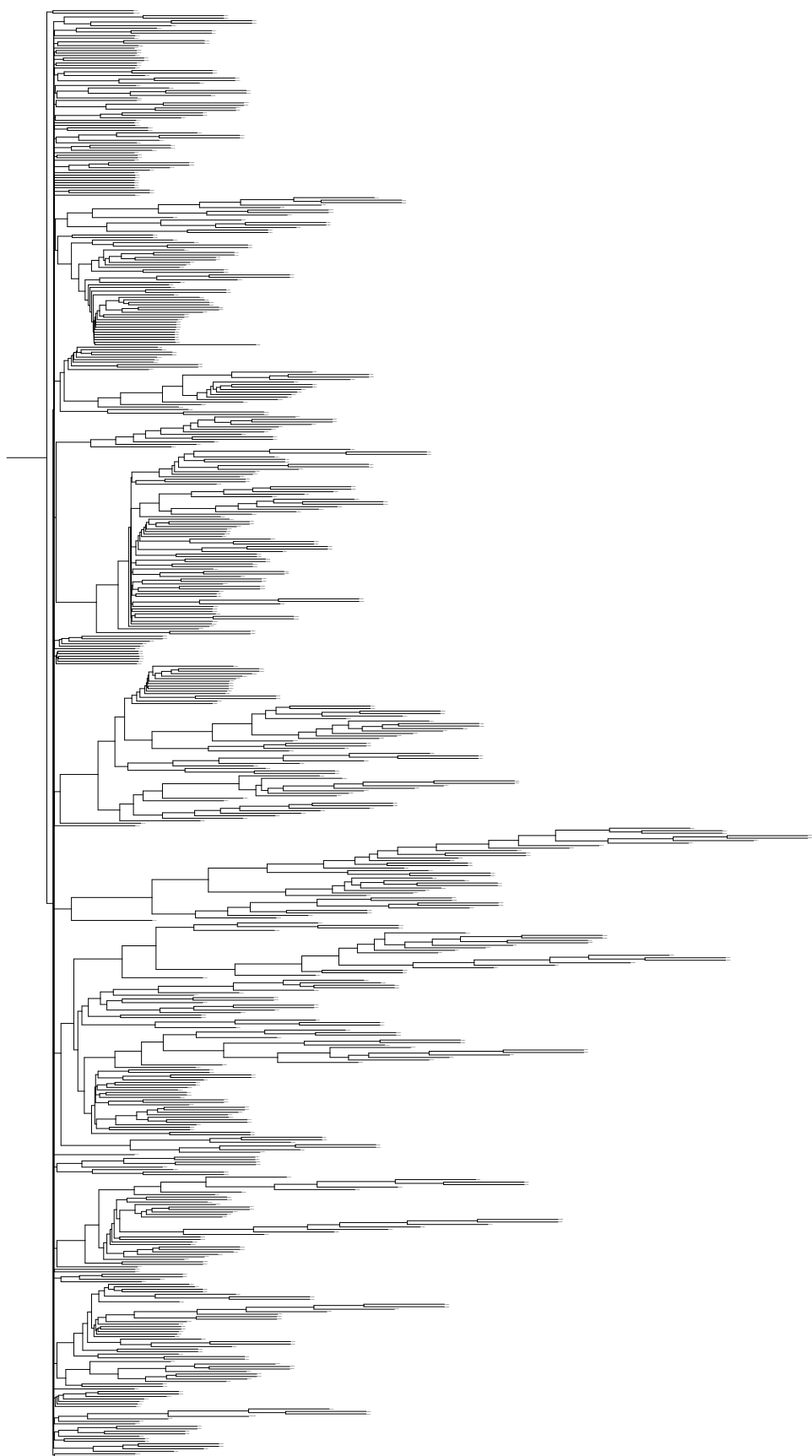

Supplement: Additional file 9 — Phylograms obtained from the distance workflow using NJ for the dataset H5N1_NA_medium.txt. [file 1471-2105-9-S1-S23-S9.pdf]

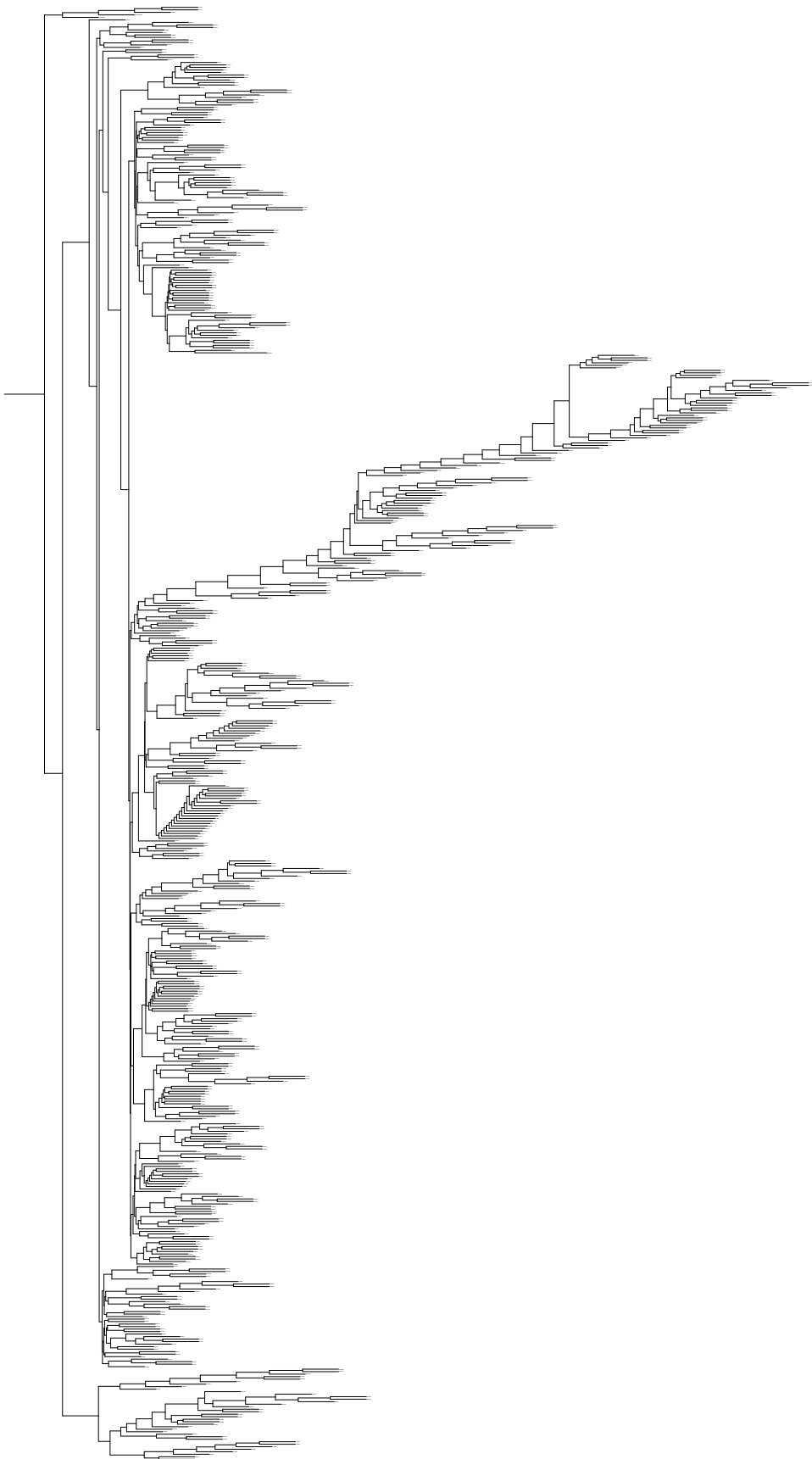

Supplement: Additional file 10 — Phylograms obtained from the parsimony workflow for the dataset H5N1_NA_medium.txt. [file 1471-2105-9-S1-S23-S10.pdf]

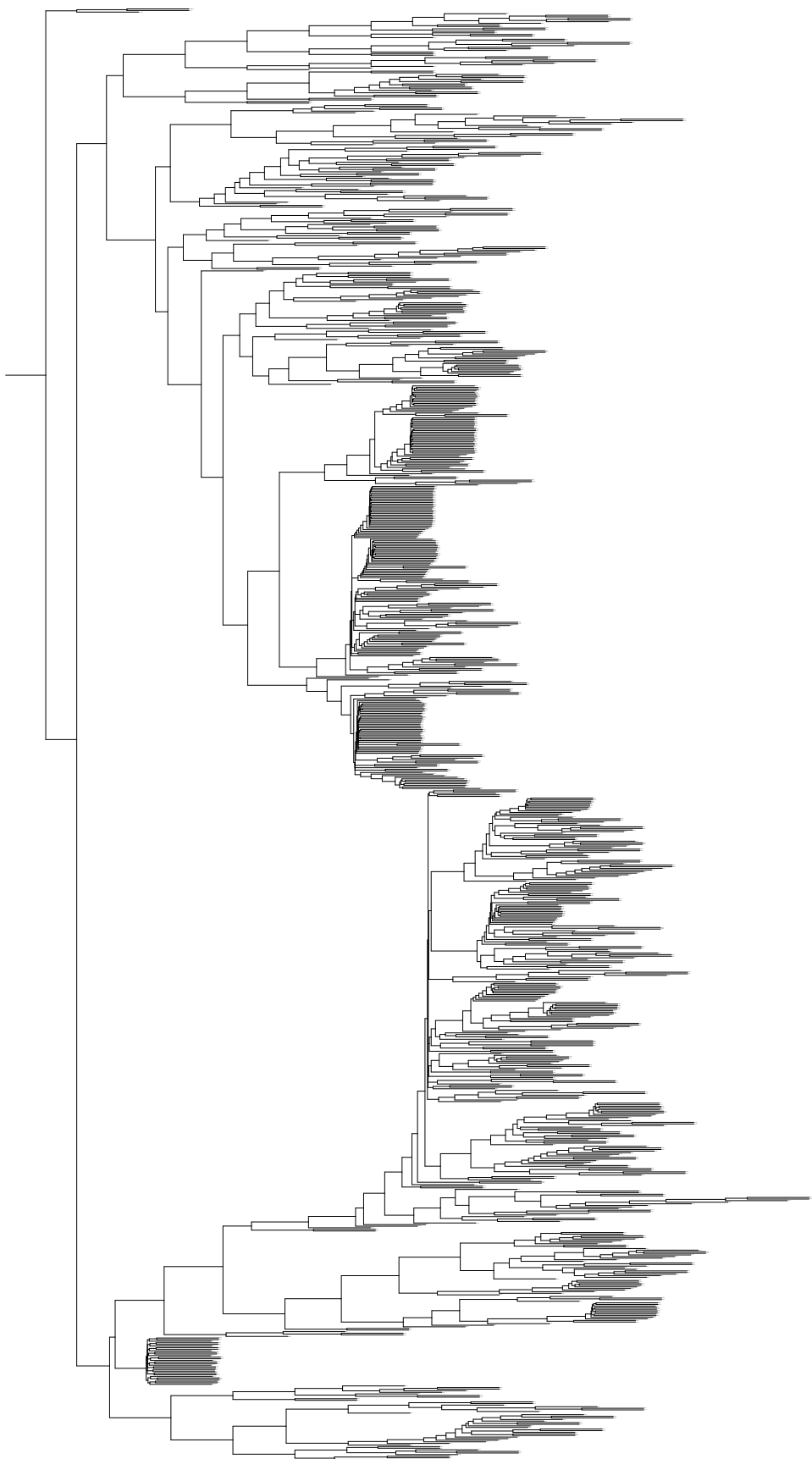

Supplement: Additional file 11 — Phylograms obtained from the distance workflow using UPGMA for the dataset H5N1_NA_macro.txt. [file 1471-2105-9-S1-S23-S11.pdf]

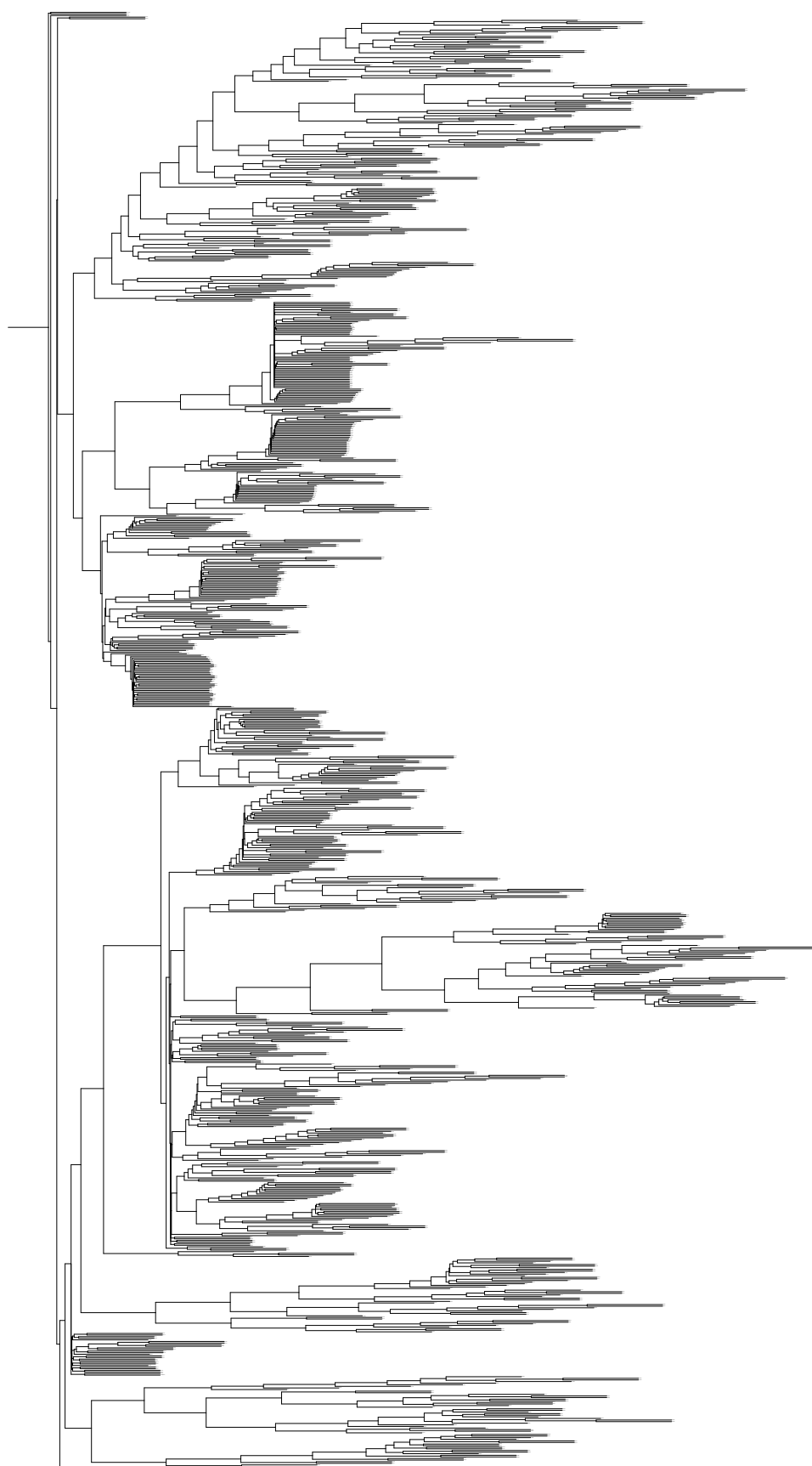

Supplement: Additional file 12 — Phylograms obtained from the distance workflow using NJ for the dataset H5N1_NA_macro.txt. [file 1471-2105-9-S1-S23-S12.pdf]

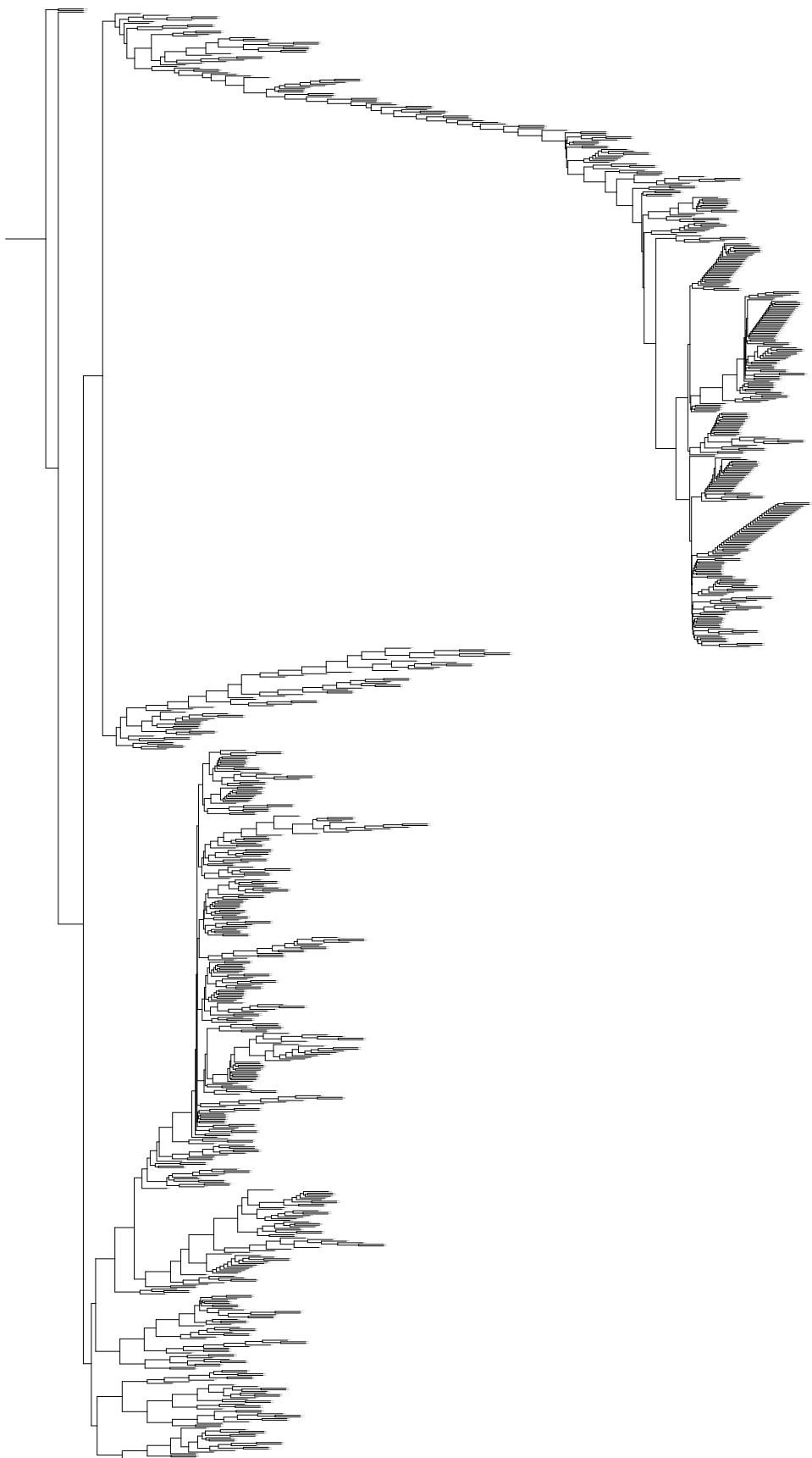

Supplement: Additional file 13 — Phylograms obtained from the parsimony workflow for the dataset H5N1_NA_macro.txt. [file 1471-2105-9-S1-S23-S13.pdf]
