# Supplementary material for: Comparative phyloinformatics of virus genes at micro and macro levels in a distributed computing environment
Source: BMC Bioinformatics. 2008 Feb 13;9(Suppl 1):S23. doi: 10.1186/1471-2105-9-S1-S23 (PMC2259424; doi:10.1186/1471-2105-9-S1-S23)
Supplement: Additional file 14 — Multiple Sequence Alignment of the Thailand clade II (Q5SDA6; Q307V5; Q6PUP6; Q5MD56) computed by ClustalW. [file 1471-2105-9-S1-S23-S14.pdf]

```
                            10        20        30        40        50        60        70          80
                   ....|....|....|....|....|....|....|....|....|....|....|....|....|....|....|   ....|....
Q6PUP6/Hu/Th/04    MNPNKKIITIGSICMVTGMVSLMLQIGNLISIWVSHSIHTGNQHKAEPISNTNFLTEKAVASVKLAGNSSLCPIN[illegible]GWAVYSKDN
Q307V5/cat/th/04   MNPNKKIITIGSICMVTGMVSLMLQIGNLISIWVSHSIHTGNQHKAEPISNTNFLTEKAVASVKLAGNSSLCPIN[illegible]GWAVYSKDN
Q5MD56/Ti/Th/04    MNPNKKIITIGSICMVTGMVSLMLQIGNLISIWVSHSIHTGNQHKAEPISNTNFLTEKAVASVKLAGNSSLCPIN[illegible]GWAVYSKDN
Q5SDA6/ch/Th/04    MNPNKKIITIGSICMVTGMVSLMLQIGNLISIWVSHSIHTGNQHKAEPISNTNFLTEKAVASVKLAGNSSLCPIN[illegible]GWAVYSKDN
```

```
                           110       120       130       140       150       160       170         180
                   ....|....|....|....|....|....|....|....|....|....|....|....|....|....|....|   ....|....
Q6PUP6/Hu/Th/04    FISCSHLECRTFFLTQGALLNDKHSNGTVKDRSPHRTLMSCPVGEAPSPYNSRFESVAWSASACHDGTSWLTIGI[illegible]SGPDNGAVA
Q307V5/cat/th/04   FISCSHLECRTFFLTQGALLNDKHSNGTVKDRSPHRTLMSCPVGEAPSPYNSRFESVAWSASACHDGTSWLTIGI[illegible]SGPDNGAVA
Q5MD56/Ti/Th/04    FISCSHLECRTFFLTQGALLNDKHSNGTVKDRSPHRTLMSCPVGEAPSPYNSRFESVAWSASACHDGTSWLTIGI[illegible]SGPDNGAVA
Q5SDA6/ch/Th/04    FISCSHLECRTFFLTQGALLNDKHSNGTVKDRSPHRTLMSCPVGEAPSPYNSRFESVAWSASACHDGTSWLTIGI[illegible]SGPDNGAVA
```

```
                           210       220       230       240       250       260       270         280
                   ....|....|....|....|....|....|....|....|....|....|....|....|....|....|....|   ....|....
Q6PUP6/Hu/Th/04    NNILRTQESECACVNGSCFTVMTDGPSNGQASHKIFKMEKGKVVKSVELDAPNYHYEECSCYPDAGEITCVCRDN[illegible]WHGSNRPWV
Q307V5/cat/th/04   NNILRTQESECACVNGSCFTVMTDGPSNGQASHKIFKMEKGKVVKSVELDAPNYHYEECSCYPDAGEITCVCRDN[illegible]WHGSNRPWV
Q5MD56/Ti/Th/04    NNILRTQESECACVNGSCFTVMTDGPSNGQASHKIFKMEKGKVVKSVELDAPNYHYEECSCYPDAGEITCVCRDN[illegible]WHGSNRPWV
Q5SDA6/ch/Th/04    NNILRTQESECACVNGSCFTVMTDGPSNGQASHKIFKMEKGKVVKSVELDAPNYHYEECSCYPDAGEITCVCRDN[illegible]WHGSNRPWV
```

```
                           310       320       330       340       350       360       370         380
                   ....|....|....|....|....|....|....|....|....|....|....|....|....|....|....|   ....|....
Q6PUP6/Hu/Th/04    VFGDNPRPNDGTGSCGPVSSNGAYGVKGFSFKYGNGVWIGRTKSTNSRSGFEMIWDPNGWTETDSSFSVKQDIVA[illegible]ITDWSGYSG
Q307V5/cat/th/04   VFGDNPRPNDGTGSCGPVSSNGAYGVKGFSFKYGNGVWIGRTKSTNSRSGFEMIWDPNGWTETDSSFSVKQDIVA[illegible]ITDWSGYSG
Q5MD56/Ti/Th/04    VFGDNPRPNDGTGSCGPVSSNGAYGVKGFSFKYGNGVWIGRTKSTNSRSGFEMIWDPNGWTETDSSFSVKQDIVA[illegible]ITDWSGYSG
Q5SDA6/ch/Th/04    VFGDNPRPNDGTGSCGPVSSNGAYGVKGFSFKYGNGVWIGRTKSTNSRSGFEMIWDPNGWTETDSSFSVKQDIVA[illegible]ITDWSGYSG
```

```
                          410       420       430       440
                  ....|....|....|....|....|....|....|....|....|....
Q6PUP6/Hu/Th/04   CFWVELIRGRPKESTIWTSGSSISFCGVNSDTVGWSWPDGAELPFTIDK
Q307V5/cat/th/04  CFWVELIRGRPKESTIWTSGSSISFCGVNSDTVGWSWPDGAELPFTIDK
Q5MD56/Ti/Th/04   CFWVELIRGRPKESTIWTSGSSISFCGVNSDTVGWSWPDGAELPFTIDK
Q5SDA6/ch/Th/04   CFWVELIRGRPKESTIWTSGSSISFCGVNSDTVGWSWPDGAELPFTIDK
```
