# Supplementary material for: Comparative phyloinformatics of virus genes at micro and macro levels in a distributed computing environment
Source: BMC Bioinformatics. 2008 Feb 13;9(Suppl 1):S23. doi: 10.1186/1471-2105-9-S1-S23 (PMC2259424; doi:10.1186/1471-2105-9-S1-S23)
Supplement: Additional file 15 — Multiple Sequence Alignment of all sequences in H5N1_NA_24.txt except the two outliers. [file 1471-2105-9-S1-S23-S15.pdf]

```
                                      10        20        30        40        50        60        70
                              ....|....|....|....|....|....|....|....|....|....|....|....|....|....|....|....
Q2LDC0/Qu/Th/06               MNPNKKIITIGSICMVTGMVSLMLQIGNLISIWVSRSIHTGNQQKAEP--------------------ISNTNFLTEKA
Q2LDC8/ch/Th/05               MNPNKKIITIGSICMVTGMVSLMLQIGNLISIWLSRSIHTGNQQKAEP--------------------ISNTNFLTEKA
Q2L700/Hu/Th/05               MNPNKKIITIGSICMVTGMVSLMLQIGNLISIWVSHSIHTGNQQKAEP--------------------ISNTNFLTEKA
Q6PUP7/Hu/Th/04               MNPNKKIITIGSICMVTGMVSLMLQIGNLISIWVSHSIHTGNQQKAEP--------------------ISNTNFLTEKA
Q307U7/Pigeon/Th/04           MNPNKKIITIGSICMVTGMVSLMLQIGNLISIWVSHSIHTGNQQKAEP--------------------ISNTNFLTEKA
Q45ZM8/wpf/th/04              MNPNKKIITIGSICMVTGMVSLMLQIGNLISIWVSHSIHTGNQHKAEP--------------------ISNTNFLTEKA
Q6B518/ch/Th/04               MNPNQKIITIGSICMVTGMVSLMLQIGNLISIWVSHSIHTGNQHKAEP--------------------ISNTNFLTEKA
Q4PKD4/ch/Th/04               MNPNQKIITIGSICMVTGMVSLMLQIGNLISIWVSHSIHTGNQHKAEP--------------------ISNTNFLTEKA
Q6PUP6/Hu/Th/04               MNPNKKIITIGSICMVTGMVSLMLQIGNLISIWVSHSIHTGNQHKAEP--------------------ISNTNFLTEKA
Q307V5/cat/th/04              MNPNKKIITIGSICMVTGMVSLMLQIGNLISIWVSHSIHTGNQHKAEP--------------------ISNTNFLTEKA
Q5MD56/Ti/Th/04               MNPNKKIITIGSICMVTGMVSLMLQIGNLISIWVSHSIHTGNQHKAEP--------------------ISNTNFLTEKA
Q5SDA6/ch/Th/04               MNPNKKIITIGSICMVTGMVSLMLQIGNLISIWVSHSIHTGNQHKAEP--------------------ISNTNFLTEKA
Q0PEF9/ch/In/06               MNPNQKIITIGSICMVTGIVSLMLQIGNMISIWVSHSIHTGNQHQAEP--------------------ISNTNFLTEKA
Q0PEG0/ch/In/06               MNPNQKIITIGSICMVTGIVSLMLQIGNMISIWVSHSIHTGNQHQAEP--------------------ISNTNFLTEKA
A1EHP3/go/Ba/06               MNPNQKIITIGSICMVIGIVSLMLQIGNMISIWVSHSIQTGNQRQAEP--------------------ISNTKFLTEKA
A1EHP1/go/Bav/06              MNPNQKIITIGSICMVIGIVSLMLQIGNMISIWVSHSIQTGNQRQAEP--------------------ISNTKFLTEKA
Q6DTU0/swine/ch/03            MNPNQKIITIGSICMVIGIVSLMLQIGNIISIWASHSIQTGNQHQAEP--------------------ISNTNFLAEKA
Q9WAA1/ch/hk97                MNPNQKIITIGSICMVVGIISLMLQIGNIISVWVSHIIQTWHPNQPEPCNQSIN-------------------FYTEQA
Q9Q0U7/gs/gd97                MNPNQKIITIGSICMVVGIISLMLQIGNIISIWVSHSIQTGNQHQAEPCNQSIITYENNTWVNQTYVNISNTNFLTEKA
Q9W7Y7/hu/hk97                MNPNQKIITIGSICMVVGIISLMLQIGNIISVWVSHIIQTWHPNQPEPCNQSIN-------------------FYTEQA
Q0A2H3/Ch/sc/59               MNPNQKIITIGSICMIVGIISLILQIGNIISIWVSHSIQTGNQNQPEICNQSIITYENNTWVNQTYVNISNTNFVTEQA
Q710U6/ch/Sc/59               MNPNQKIITIGSICMIVGIISLILQIGNIISIWVSHSIQTGNQNQPEICNQSIITYENNTWVNQTYVNISNTNFVTEQA
```

```
                               110       120       130       140       150       160       170
                           ....|....|....|....|....|....|....|....|....|....|....|....|....|....|....
Q2LDC0/Qu/Th/06            SKDNSIRIGSKGDVFVIREPFISCSHLECRTFFLTQGSLLNDKHSNGTVKDRSPHRTLMSCPVGEAPSPYNSRFESVAW
Q2LDC8/ch/Th/05            SKDNSIRIGSKGDVFVIREPFISCSHLECRTFFLTQGSLLNDKHSNGTVKDRSPHRTLMSCPVGEAPSPYNSRFESVAW
Q2L700/Hu/Th/05            SKDNSIRIGSKGDVFVIREPFISCSHLECRTFFLTQGSLLNDKHSNGTVKDRSPHRTLMSCPVGEAPSPYNSRFESVAW
Q6PUP7/Hu/Th/04            SKDNSIRIGSKGDVFVIREPFISCSHLECRTFFLTQGALLNDKHSNGTVKDRSPHRTLMSCPVGEAPSPYNSRFESVAW
Q307U7/Pigeon/Th/04        SKDNSIRIGSKGDVFVIREPFISCSHLECRTFFLTQGALLNDKHSNGTVKDRSPHRTLMSCPVGEAPSPYNSRFESVAW
Q45ZM8/wpf/th/04           SKDNSIRIGSKGDVFVIREPFISCSHLECRTFFLTQGALLNDKHSNGTVKDRSPHRTLMSCPVGEAPSPYNSRFESVAW
Q6B518/ch/Th/04            SKDNSIRIGSKGDVFVIREPFISCSHLECRTFFLTQGALLNDKHSNGTVKDRSPHRTLMSCPVGEAPSPYNSRFESVAW
Q4PKD4/ch/Th/04            SKDNSIRIGSKGDVFVIREPFISCSHLECRTFFLTQGALLNDEHSNGTVKDRSPHRTLMSCPVGEAPSPYNSRFESVAW
Q6PUP6/Hu/Th/04            SKDNSIRIGSKGDVFVIREPFISCSHLECRTFFLTQGALLNDKHSNGTVKDRSPHRTLMSCPVGEAPSPYNSRFESVAW
Q307V5/cat/th/04           SKDNSIRIGSKGDVFVIREPFISCSHLECRTFFLTQGALLNDKHSNGTVKDRSPHRTLMSCPVGEAPSPYNSRFESVAW
Q5MD56/Ti/Th/04            SKDNSIRIGSKGDVFVIREPFISCSHLECRTFFLTQGALLNDKHSNGTVKDRSPHRTLMSCPVGEAPSPYNSRFESVAW
Q5SDA6/ch/Th/04            SKDNSIRIGSKGDVFVIREPFISCSHLECRTFFLTQGALLNDKHSNGTVKDRSPHRTLMSCPVGEAPSPYNSRFESVAW
Q0PEF9/ch/In/06            SKDNSIRIGSKGDVFVIREPFISCSHLECRTFFLTQGALLNDKHSNGTVKDRSPHRTLMSCPVGVAPSPYNSRFESVAW
Q0PEG0/ch/In/06            SKDNSIRIGSKGDVFVIREPFISCSHLECRTFFLTQGALLNDKHSNGTVKDRSPHRTLMSCPVGVAPSPYNSRFESVAW
A1EHP3/go/Ba/06            SKDNSIRIGSRGDVFVIREPFISCSHLECRTFFLTQGALLNDKHSNGTIKDRSPHRTLMSCPVGEAPSPYNSRFESVAW
A1EHP1/go/Bav/06           SKDNSIRIGSRGDVFVIREPFISCSHLECRTFFLTQGALLNDKHSNGTIKDRSPHRTLMSCPVGEAPSPYNSRFESVAW
Q6DTU0/swine/ch/03         SKDNGIRIGSKGDVFVIREPFISCSHLECRTFFLTQGALLNDKHSNGTVKDRSPHRALMSCPVGEAPSPYNSRFESVAW
Q9WAA1/ch/hk97             SKDNSIRIGSKGDVFVIREPFISCSHLECRTFFLTQGALLNDKHSNGTVKDRSPYRTLMSCPVGEAPSPYKSRFESVAW
Q9Q0U7/gs/gd97             SKDNGIRIGSKGDVFVIREPFISCSHLECRTFFLTQGALLNDKHSNGTVKDRSPHRTLMSCPVGEAPSPYNSRFESVAW
Q9W7Y7/hu/hk97             SKDNSIRIGSKGDVFVIREPFISCSHLECRTFFLTQGALLNDKHSNGTVKDRSPYRTLMSCPVGEAPSPYNSRFESVAW
Q0A2H3/Ch/sc/59            SKDNGIRIGSKGDVFVIREPFISCSHLECRTFFLTQGALLNDKHSNGTVKDRSPYRTLMSCPIGESPSPYNSRFESVAW
Q710U6/ch/Sc/59            SKDNGIRIGSKGDVFVIREPFISCSHLECRTFFLTQGALLNDKHSNGTVKDRSPYRTLMSCPIGESPSPYNSRFESVAW
```

```
                                 210       220       230       240       250       260       270       2
                             ....|....|....|....|....|....|....|....|....|....|....|....|....|.. ..|....
Q2LDC0/Qu/Th/06              GAVAVLKYNGIITDTIKSWRNNILRTQESECACVNGSCFTVMTDGPSNGQASHKIFKMEKGKVVKSVELDAPNYHYEEC
Q2LDC8/ch/Th/05              GAVAVLKYNGIITDTIKSWRNNILRTQESECACVNGSCFTVMTDGPSNGQASHKIFKMEKGKVVKSVELDAPNYHYEEC
Q2L700/Hu/Th/05              GAVAVLKYNGIITDTIKSWRNNILRTQESECACVNGSCFTVMTDGPSNGQASHKIFKMDKGKVVKSVELDAPNYHYEEC
Q6PUP7/Hu/Th/04              GAVAVLKYNGIITDTIKSWRNNILRTQESECACVNGSCFTVMTDGPSNGQASHKIFKMEKGKVVKSVELDAPNYHYEEC
Q307U7/Pigeon/Th/04          GAVAVLKYNGIITDTIKSWRNNILRTQESECACVNGSCFTVMTDGPSNGQASHKIFKMEKGKVVKSVELDAPNYHYEEC
Q45ZM8/wpf/th/04             GAVAVLKYNGIITDTIKSWRNNILRTQESECACVNGSCFTVMTDGPSNGQASHKIFKMEKGKVVKSVELDAPNYHYEEC
Q6B518/ch/Th/04              GAVAVLKYNGIITDTIKSWRNNILRTQESECACVNGSCFTVMTDGPSNGQASHKIFKMEKGKVVKSVELDAPNYHYEEC
Q4PKD4/ch/Th/04              GAVAVLKYNGIITDTIKSWRNNILRTQESECACVNGSCFTVMTDGPSNGQASHKIFKMEKGKVVKSVELDAPNYHYEEC
Q6PUP6/Hu/Th/04              GAVAVLKYNGIITDTIKSWRNNILRTQESECACVNGSCFTVMTDGPSNGQASHKIFKMEKGKVVKSVELDAPNYHYEEC
Q307V5/cat/th/04             GAVAVLKYNGIITDTIKSWRNNILRTQESECACVNGSCFTVMTDGPSNGQASHKIFKMEKGKVVKSVELDAPNYHYEEC
Q5MD56/Ti/Th/04              GAVAVLKYNGIITDTIKSWRNNILRTQESECACVNGSCFTVMTDGPSNGQASHKIFKMEKGKVVKSVELDAPNYHYEEC
Q5SDA6/ch/Th/04              GAVAVLKYNGIITDTIKSWRNNILRTQESECACVNGSCFTVMTDGPSNGQASHKIFKMEKGKVVKSVELDAPNYHYEEC
Q0PEF9/ch/In/06              GAVAVLKYNGIITDTIKSWRNNILRTQESECACVNGSCFTVMTDGPSNGQASHKIFKMEKGKVVKSVELDAPNYHYEEC
Q0PEG0/ch/In/06              GAVAVLKYNGIITDTIKSWRNNILRTQESECACVNGSCFTVMTDGPSNGQASHKIFKMEKGKVVKSVELDAPNYHYEEC
A1EHP3/go/Ba/06              GAVAVLKYNGIITDTIKSWRNNILRTQESECACVNGSCFTVMTDGPSSGQASYKIFKMEKGKVVKSVELDAPNYHYEEC
A1EHP1/go/Bav/06             GAVAVLKYNGIITDTIKSWRNNILRTQESECACVNGSCFTVMTDGPSSGQASYKIFKMEKGKVVKSVELDAPNYHYEEC
Q6DTU0/swine/ch/03           GAVAVLKYNGIITDTIKSWRNNILRTQESECACVNGSCFTVMTDGPSNGQASYKIFKMEKGKVVKSVELDAPNYHYEEC
Q9WAA1/ch/hk97               GAVAVLKYNGIITDTIKSWRNNILRTQESECACVNGSCFTVMTDGPSNEQASYKIFKIEKGRVVKSVELNAPNYHYEEC
Q9Q0U7/gs/gd97               GAVAVLKYNGIITDTIKSWRNNILRTQESECACVNGSCFTVMTDGPSNGQASYKIFKMEKGKVVKSVELNAPNYHYEEC
Q9W7Y7/hu/hk97               GAVAVLKYNGIITDTIKSWRNNILRTQESECACVNGSCFTVMTDGPSNEQASYKIFKIEKGRVVKSVELNAPNYHYEEC
Q0A2H3/Ch/sc/59              GAVAVLKYNGIITDTIKSWRNNILRTQESECACMNGSCFTIMTDGPSNGQASYKIFKIEKGKVVKSVELNAPNYHYEEC
Q710U6/ch/Sc/59              GAVAVLKYNGIITDTIKSWRNNILRTQESECACMNGSCFTIMTDGPSNGQASYKIFKIEKGKVVKSVELNAPNYHYEEC
```

```
                                    310       320       330       340       350       360       370       380
                               ....|....|....|....|....|....|....|....|....|....|....|....|....|....|....|....|.
Q2LDC0/Qu/Th/06                RPWVSFNQNLEYQIGYICSGVFGDTPRPNDGTGSCGPVSSNGAYGVKGFSFKYGNGVWIGRTKSTNSRSGFEMIWDPNGWT
Q2LDC8/ch/Th/05                RPWVSFNQNLEYQIGYICSGVFGDTPRPNDGTGSCGPVSSNGTYGVKGFSFKYGNGVWIGRTKSTNSRSGFEMIWDPNGWT
Q2L700/Hu/Th/05                RPWVSFNQNLEYQIGYICSGVFGDTPRPNDGTGSCGPVSSNGTYGVKGFSFKYGNGVWIGRTKSTNSRSGFEMIWDPNGWT
Q6PUP7/Hu/Th/04                RPWVSFNQNLEYQIGYICSGVFGDNPRPNDGTGSCGPVSSNGAYGVKGFSFKYGNGVWIGRTKSTNSRSGFEMIWDPNGWI
Q307U7/Pigeon/Th/04            RPWVSFNQNLEYQIGYICSGVFGDNPRPNDGTGSCGPVSSNGAYGVKGFSFKYGNGVWIGRTKSTNSRSGFEMIWDPNGWT
Q45ZM8/wpf/th/04               RPWVSFNQNLEYQIGYICSGVFGDNPRPNDGTGSCGPVSSNGAYGVKGFSFKYGNGVWIGRTKSTNSRSGFEMIWDPNGWT
Q6B518/ch/Th/04                RPWVSFNQNLEYQIGYICSGVFGDNPRPNDGTGSCGPVSSNGAYGVKGFSFKYGNGVWIGRTKSTNSRSGFEMIWDPNGWT
Q4PKD4/ch/Th/04                RPWVSFNQNLEYQIGYICSGVFGDYPRPNDGTGSCGPVSSNGAYGVKGFSFNHGNGVWIGRTKSTNSRSGFEMIWGPNGWT
Q6PUP6/Hu/Th/04                RPWVSFNQNLEYQIGYICSGVFGDNPRPNDGTGSCGPVSSNGAYGVKGFSFKYGNGVWIGRTKSTNSRSGFEMIWDPNGWT
Q307V5/cat/th/04               RPWVSFNQNLEYQIGYICSGVFGDNPRPNDGTGSCGPVSSNGAYGVKGFSFKYGNGVWIGRTKSTNSRSGFEMIWDPNGWT
Q5MD56/Ti/Th/04                RPWVSFNQNLEYQIGYICSGVFGDNPRPNDGTGSCGPVSSNGAYGVKGFSFKYGNGVWIGRTKSTNSRSGFEMIWDPNGWT
Q5SDA6/ch/Th/04                RPWVSFNQNLEYQIGYICSGVFGDNPRPNDGTGSCGPVSSNGAYGVKGFSFKYGNGVWIGRTKSTNSRSGFEMIWDPNGWT
Q0PEF9/ch/In/06                RPWVSFNQNLEYQIGYICSGVFGDNPRPNDGTGSCGPVSSNGAYGVKGFSFRYGNGVWIGRTKSTNSRSGFEMIWDPNGWT
Q0PEG0/ch/In/06                RPWVSFNQNLEYQIGYICSGVFGDNPRPNDGTGSCGPVSSNGAYGVKGFSFRYGNGVWIGRTKSTNSRSGFEMIWDPNGWT
A1EHP3/go/Ba/06                RPWVSFNQNLEYQIGYICSGVFGDNPRPNDGTGSCGPVSPNGAYGVKGFSFKYGNGVWIGRTKSTNSRSGFEMIWDPNGWT
A1EHP1/go/Bav/06               RPWVSFNQNLEYQIGYICSGVFGDNPRPNDGTGSCGPVSPNGAYGVKGFSFKYGNGVWIGRTKSTNSRSGFEMIWDPNGWT
Q6DTU0/swine/ch/03             RPWVSFNQNLEYQIGYICSGVFGDNPRPNDGTGSCGPVSPNGAYGVKGFSFKYGNGVWIGRTKSINSRSGFEMIWDPNGWT
Q9WAA1/ch/hk97                 RPWVSFNQNLEYQIGYICSGVFGDSPRPNDGTGSCGPVSLNGAYGVKGFSFKYGNGVWIGRTKSTSSRSGFEMIWDPNGWT
Q9Q0U7/gs/gd97                 RPWVSFNQNLEYQIGYICSGVFGDNPRPNDGTGSCGPVSPNGAYGVKGFSFKYGNGVWIGRTKSTNSRSGFEMIWDPNGWT
Q9W7Y7/hu/hk97                 RPWVSFNQNLEYQIGYICSGVFGDSPRPNDGTGSCGPVSLNGAYGVKGFSFKYGNGVWIGRTKSTSSRSGFEMIWDPNGWT
Q0A2H3/Ch/sc/59                RPWVSFNQNLEYQIGYICSGVFGDNPRPNDGAGSCGPVSSNGAYGVKGFSFKYGKGVWIGRTKSTSSRSGFEMIWDPNGWT
Q710U6/ch/Sc/59                RPWVSFNQNLEYQIGYICSGVFGDNPRPNDGAGSCGPVSSNGAYGVKGFSFKYGKGVWIGRTKSTSSRSGFEMIWDPNGWT
```

```
                                 410       420       430       440       450       460
                        ....|....|....|....|....|....|....|....|....|....|....|....|....|....
Q2LDC0/Qu/Th/06         GYSGSFVQHPELTGLDCIRPCFWVELIRGRPKESTIWTSGSSISFCGVNSDTVGWSWPDGAELPFTIDK
Q2LDC8/ch/Th/05         GYSGSFVQHPELTGLNCIRPCFWVELIRGRPKESTIWTSGSSISFCGVNSDTVGWSWPDGAELPFTIDK
Q2L700/Hu/Th/05         GYSGSFVQHPELTGLDCIRPCFWVELIRGRPKESTIWTSGSSISFCGVNSDTVGWSWPDGAELPFTIDK
Q6PUP7/Hu/Th/04         GYSGSFVQHPELTGLDCIRPCFWVELIRGRPKESTIWTSGSSISFCGVNSDTVGWSWPDGAELPFTIDK
Q307U7/Pigeon/Th/04     GYSGSFVQHPELTGLDCIRPCFWVELIRGRPKESTIWTSGSSISFCGVNSDTVGWSWPDGAELPFTIDK
Q45ZM8/wpf/th/04        GYSGSFVQHPELTGLDCIRPCFWVELIRGRPKESTIWTSGSSISFCGVNSDTVGWSWPDGAELPFTIDN
Q6B518/ch/Th/04         GYSGSFVQHPELTGLDCIRPCFWVELIRGRPKESTIWTSGSSISFCGVNSDTVGWSWPDGAELPFTIDK
Q4PKD4/ch/Th/04         GYSGSFVQHPELTGLDCIRPCFWVELIRGRPKESTIWTSGSSISFCGVNSDTVGWSWPDGAELPFTIDK
Q6PUP6/Hu/Th/04         GYSGSFVQHPELTGLDCIRPCFWVELIRGRPKESTIWTSGSSISFCGVNSDTVGWSWPDGAELPFTIDK
Q307V5/cat/th/04        GYSGSFVQHPELTGLDCIRPCFWVELIRGRPKESTIWTSGSSISFCGVNSDTVGWSWPDGAELPFTIDK
Q5MD56/Ti/Th/04         GYSGSFVQHPELTGLDCIRPCFWVELIRGRPKESTIWTSGSSISFCGVNSDTVGWSWPDGAELPFTIDK
Q5SDA6/ch/Th/04         GYSGSFVQHPELTGLDCIRPCFWVELIRGRPKESTIWTSGSSISFCGVNSDTVGWSWPDGAELPFTIDK
Q0PEF9/ch/In/06         GYSGSFVQHPELTGLDCIRPCFWVELIRGRPKESTIWTSGSSISFCGVNSDTVGWSWPDGAELPFTIDK
Q0PEG0/ch/In/06         GYSGSFVQHPELTGLDCIRPCFWVELIRGRPKESTIWTSGSSISFCGVNSDTVGWSWPDGAELPFTIDK
A1EHP3/go/Ba/06         GYSGSFVQHPELTGLDCIRPCFWVELIRGRPKESTIWTSGSSISFCGVNSDTVSWSWPDGAELPFTIDK
A1EHP1/go/Bav/06        GYSGSFVQHPELTGLDCIRPCFWVELIRGRPKESTIWTSGSSISFCGVNSDTVSWSWPDGAELPFTIDK
Q6DTU0/swine/ch/03      GYSGSFVQHPELTGLDCIRPCFWVELIRGRPKESTIWTSGSSISFCGVNSDTVGWSWPDGAELPFTIDK
Q9WAA1/ch/hk97          GYSGSFIQHPELTGLNCMRPCFWVELIRGRPKEKTIWTSGSSISFCGVNSDTVGWSWPDGAELPFTIDK
Q9Q0U7/gs/gd97          GYSGSFVQHPELTGLDCIRPCFWVELIRGRPKESTIWTSGSSISFCGVNSDTVGWSWPDDAELPFTIDK
Q9W7Y7/hu/hk97          GYSGSFIQHPELTGLNCMRPCFWVELIRGRPKEKTIWTSGSSISFCGVNSDTVGWSWPDGADLPFTIDK
Q0A2H3/Ch/sc/59         GYSGSFVQHPELTGLDCMRPCFWVELIRGRPKENTIWTSGSSISFCGVNSDTVGWSWPDGAELPFTIDK
Q710U6/ch/Sc/59         GYSGSFVQHPELTGLDCMRPCFWVELIRGRPKENTIWTSGSSISFCGVNSDTVGWSWPDGAELPFTIDK
```
